# Development and validation of subtype-specific simplified ultrasound assessment systems for juvenile idiopathic arthritis: a prospective observational study

**DOI:** 10.3389/fped.2026.1876983

**Published:** 2026-07-06

**Authors:** Lirong Zhu, Juan Wang, Chunmei Yin, Chunjiang Yang, Min Qiu, Xuemei Tang, Huan Xiao

**Affiliations:** 1 Department of Ultrasound, Children's Hospital of Chongqing Medical University, National Clinical Research Center for Children and Adolescents' Health and Diseases, Ministry of Education Key Laboratory of Child Development and Disorders, Chongqing, China; 2 Chongqing Key Laboratory of Child Rare Diseases in Infection and Immunity, Children’s Hospital of Chongqing Medical University, Chongqing, China; 3 International Science and Technology Cooperation Base of Child Development and Critical Disorders, Children’s Hospital of Chongqing Medical University, Chongqing, China; 4 Department of Ultrasound Medicine, School of Medicine, Renji Hospital, Chongqing University, Chongqing, China; 5 Department of Ultrasound, Chongqing YouYou BaoBei Women’s and Children’s Hospital, Chongqing, China; 6 Department of Rheumatology and Immunology, Children's Hospital of Chongqing Medical University, National Clinical Research Center for Children and Adolescents' Health and Diseases, Ministry of Education Key Laboratory of Child Development and Disorders, Children’s Hospital of Chongqing Medical University, Chongqing, China

**Keywords:** disease activity, juvenile idiopathic arthritis, musculoskeletal diseases, oligoarticular, polyarticular, synovitis, ultrasonography

## Abstract

**Objectives:**

Comprehensive ultrasound effectively assesses synovitis in juvenile idiopathic arthritis (JIA) but is time-consuming. Existing simplified scores apply a uniform joint combination to all subtypes, overlooking the differences between the oligoarticular (oJIA) and polyarticular (pJIA) forms. We aimed to develop subtype-specific simplified ultrasound systems to improve clinical efficiency.

**Methods:**

In this prospective study, 83 patients with active oJIA (*n* = 42) or pJIA (*n* = 41) underwent a standardized 68-joint grayscale and Power Doppler assessment. Subtype-specific models were constructed using data-driven stepwise selection to ensure 100% patient coverage. Primary endpoints were diagnostic performance for high disease activity (according to the Juvenile Arthritis Disease Activity Score-27, JADAS27) and numerical agreement.

**Results:**

Examination time was reduced by approximately 67% (∼13 vs. 38.8 min). The oJIA model required 4 joint pairs (elbows, knees, ankles, MCP1); although its correlation with systemic activity was limited by localized disease, it showed excellent numerical agreement with the comprehensive score, supporting its use for monitoring. The pJIA model (6 joint pairs: elbows, wrists, hips, knees, PIP3, PIP5) showed superior diagnostic performance for high disease activity (AUC = 0.88) compared with the all-patient model (AUC = 0.67), and a strong correlation with JADAS27 (rs = 0.68, *P* < 0.001).

**Conclusions:**

Subtype-specific simplified ultrasound systems substantially reduce examination time while maintaining accuracy. The pJIA model effectively grades disease activity, whereas the oJIA model provides a reliable, efficient tool for longitudinal monitoring. This individualized approach optimizes workflow efficiency in pediatric rheumatology.

## Highlights

- Subtype-specific simplified ultrasound models were developed to address the distinct joint involvement patterns in JIA.
- These novel models reduced the examination time by approximately 67% compared to comprehensive scanning.
- The polyarticular JIA model (6 joint pairs) demonstrates excellent diagnostic performance for detecting high disease activity.
- The oligoarticular JIA model (4 joint pairs) shows high numerical agreement suitable for longitudinal disease monitoring.

## Introduction

Juvenile idiopathic arthritis (JIA), the most common chronic rheumatic disease of childhood, has a global annual incidence of approximately 1.6–23 per 100,000, with marked epidemiological variation across regions and populations (1, 2). Accurate assessment of disease activity is essential for early intervention, treatment optimization, and reduction of the risk of permanent joint damage (3). However, conventional assessment based on clinical examination and laboratory markers has important limitations in JIA. Acute-phase reactants such as ESR and CRP are frequently normal in oligoarticular JIA despite active disease (4), yet may remain elevated in clinically inactive disease (5). Clinical joint examination is also challenging in children, in whom limited symptom reporting and examiner dependence contribute to poor inter-examiner agreement (6) and substantial underdetection of active synovitis (7).

The value of musculoskeletal ultrasound in assessing JIA disease activity is now widely recognized. Ultrasound detects subclinical synovitis in approximately 30% of joints—substantially more than clinical examination (7)—and ultrasound scores correlate moderately with disease activity indices (rs = 0.44–0.61) (7–9). Power Doppler (PD) signal has been associated with disease activity, although its value for predicting relapse remains debated (10–12). Comprehensive ultrasound assessment, however, requires scanning of multiple joints and is time-consuming, which limits its routine use in pediatric practice (13). To address this, several simplified protocols have been developed: the 10-joint model of Collado et al. achieved 100% detection of grayscale synovitis (14), and the MUSICAL system achieved efficient synovitis coverage using five joint pairs (15). A critical limitation of these approaches, however, is that they apply a uniform joint combination to all subtypes, disregarding the substantial differences in joint involvement between JIA subtypes.

JIA subtypes differ markedly in their patterns of joint involvement and underlying pathobiology (16, 17). Oligoarticular JIA (oJIA) predominantly affects the large joints of the lower limbs, whereas polyarticular JIA (pJIA) involves a wider distribution, including small joints and tendon sheaths (18, 19). Because ultrasound can detect subclinical involvement that influences subtype classification and treatment decisions (9, 20), these subtype-specific differences are clinically important—yet they have not been adequately addressed in existing simplified protocols.

We therefore aimed to develop subtype-specific simplified ultrasound assessment systems for the two most common JIA subtypes, oJIA and pJIA. We hypothesized that, by reflecting the characteristic joint involvement of each subtype, a subtype-specific approach would yield higher diagnostic performance and provide a sound basis for precise assessment and individualized treatment.

## Materials and methods

### Study design and participants

This single-center, prospective observational study was conducted between November 2023 and October 2024 at the Department of Rheumatology and Immunology, Children's Hospital of Chongqing Medical University. We enrolled patients aged 1–16 years with active oJIA or pJIA fulfilling the International League of Associations for Rheumatology (ILAR) criteria (21).

Inclusion criteria were: (1) age 1–16 years at enrollment; (2) a confirmed diagnosis of oJIA or pJIA according to the ILAR criteria (Edmonton revision, 2001); (3) active disease at enrollment, defined as at least one joint with clinical arthritis (swelling, or limited motion accompanied by pain or tenderness); and (4) a clinical indication to initiate or adjust disease-modifying antirheumatic drug (DMARD) therapy. Exclusion criteria were: (1) a concurrent autoimmune or systemic rheumatic disease (e.g., systemic lupus erythematosus or juvenile dermatomyositis); (2) joint infection or acute joint trauma within the preceding 3 months; (3) systemic or intra-articular corticosteroid treatment within 4 weeks before enrollment; and (4) inability to complete the standardized ultrasound protocol owing to pain, distress, or lack of cooperation.

The minimum required sample size, based on pilot data and previous simplified-model studies (14, 15), was 35 patients per group to detect a moderate effect size (Cohen's d = 0.5; α = 0.05, power = 0.80). The protocol was approved by the Institutional Ethics Committee of the Children's Hospital of Chongqing Medical University [Approval No. (2023) Lunshen (Yan) 490], and written informed consent was obtained from all patients and their legal guardians.

### Clinical assessment

Clinical assessment was performed by experienced pediatric rheumatologists blinded to the ultrasound findings. Baseline data included demographic characteristics, disease duration, and serological markers [rheumatoid factor [RF] and antinuclear antibody [ANA]]. Disease activity was measured using the Juvenile Arthritis Disease Activity Score-27 (JADAS27) (22) and stratified according to the criteria of Consolaro et al. (23): high disease activity was defined as JADAS27 > 4.2 in oJIA and >8.5 in pJIA. Ten patients underwent a 3-month follow-up assessment to evaluate treatment-response sensitivity.

### Ultrasound examination

Ultrasound examination was performed using GE Logiq 11 and Logiq e systems (GE Healthcare, Milwaukee, WI, USA) with high-frequency linear array probes (7–18 MHz). Grayscale (GS) and PD techniques were applied, with PD parameters standardized to a pulse repetition frequency of 0.4–0.6 kHz, color gain set to just suppress background noise, and a low wall filter. A standardized 68-joint protocol was used, following the joint-specific scanning and scoring system of Vega-Fernandez et al. (15), with joint coverage expanded to include the bilateral shoulder, elbow, wrist, metacarpophalangeal (MCP), interphalangeal (PIP), hip, knee, ankle, and toe joints to ensure comprehensive assessment. Detailed scanning protocols and representative grading images (grayscale 0–3, Power Doppler 0–3) across pediatric age groups are provided in Supplementary Materials S1, S2, respectively.

Synovitis was defined according to the pediatric OMERACT ultrasound criteria (24), as a GS score ≥ 2 and/or a combined GS ≥ 1 plus PD ≥ 1 (7). In our cohort, all observed tenosynovitis occurred together with adjacent joint synovitis; therefore, only synovitis scores were used in the construction of the simplified joint models.

To assess the reliability of ultrasound scoring, inter-observer agreement was evaluated by two independent pediatric musculoskeletal ultrasound physicians (each with >10 years of experience), who performed blinded re-evaluation of 150 randomly selected still images (75 grayscale, 75 Power Doppler) from 40 complete examinations (40 patients). Images were selected to represent all joint types and the full range of severity (GS grades 0–3, PD grades 0–3), and were scored using the joint-specific semiquantitative system. Intraclass correlation coefficients (ICCs) were calculated with 95% confidence intervals. Inter-observer reliability was excellent for both grayscale synovitis (ICC = 0.87, 95% CI: 0.78–0.95) and Power Doppler signal (ICC = 0.85, 95% CI: 0.78–0.91), with joint-specific ICCs ranging from 0.85 to 0.93 (Supplementary Table S1).

### Construction of the simplified assessment systems

Two indicators were defined for model construction: (1) the Ultrasound Synovitis Count—the number of joints with synovitis (GS ≥ 2, or GS ≥ 1 plus PD ≥ 1), with bilateral joints counted separately; and (2) the Ultrasound Synovitis Index—the sum of all GS and PD scores across the assessed joints. Subtype-specific simplified models were constructed using a data-driven stepwise selection strategy (14, 15). The frequency of involvement was calculated for each joint within each subtype, and joints were then added sequentially by forward selection, starting from the most frequently involved, until 100% patient coverage was achieved.

### Statistical analysis

Analyses were performed using IBM SPSS Statistics 26.0 and Python 3.8. Continuous variables were tested for normality with the Shapiro–Wilk test; normally distributed data are presented as mean ± standard deviation and compared using the independent-samples *t*-test, and non-normally distributed data as median [interquartile range] and compared using the Mann–Whitney *U*-test. Categorical variables are presented as frequency (percentage) and compared using the *χ*^2^ or Fisher's exact test. Receiver operating characteristic (ROC) analysis was used to assess diagnostic performance, and Bland-Altman analysis to assess numerical agreement. For the oJIA simplified model, ROC analysis was based on the grayscale score alone, as Power Doppler signals were absent in most oJIA patients with low disease activity. Decision curve analysis was used to evaluate net clinical benefit. Spearman correlation was used to assess the relationship between ultrasound scores and clinical indicators (|rs| < 0.3, weak; 0.3–0.7, moderate; >0.7, strong). The standardized mean response (SMR) was used to assess treatment-response sensitivity (25). Comparisons of simplified model scores between high- and low-disease-activity groups were performed using the Mann–Whitney *U*-test, and Bonferroni correction was applied for multiple comparisons; *P* < 0.05 was considered statistically significant. The SMR was calculated from paired first and last visits; 10 patients had unambiguous paired ultrasound assessments.

## Results

### Patient characteristics and subtype-specific disease patterns

The study included 83 patients with active JIA—42 (50.6%) with oJIA and 41 (49.4%) with pJIA. The cohort was 61.4% female, with a median age of 8.0 [4.5, 11.5] years and a median disease duration of 25.0 [11.0, 40.0] months. The two groups did not differ significantly in sex or age but showed important differences in disease severity and serological profile. The pJIA group had higher disease activity, with significantly higher JADAS27 scores [6.1 (3.0, 9.0) vs. 4.0 (2.0, 5.4), *P* = 0.005] and active joint counts [2.0 (0.8, 4.0) vs. 1.0 (0.0, 1.3), *P* = 0.002] than the oJIA group. The pJIA group also had a higher RF-positivity rate (19.5% vs. 0.0%, *P* = 0.002), whereas ANA positivity did not differ between groups (17.1% vs. 14.3%, *P* = 1.000). These baseline differences supported the development of subtype-specific assessment strategies (Supplementary Table S2).

### Joint involvement: ultrasound vs. clinical examination

Overall agreement between ultrasound and clinical examination was slight (Kappa = 0.16, *P* < 0.001), with substantial variation across joints (range: −0.03 to 0.48); concordance was highest at the wrist (Kappa = 0.48) and lowest at the hip (Kappa = 0.09). Ultrasound detected higher synovitis rates than clinical examination at the elbow (27.7% vs. 8.4%), knee (31.3% vs. 25.3%), PIP (18.1% vs. 4.8%), and MCP (12.0% vs. 4.8%) joints. Thirty patients (36.1%) were ultrasound-negative across all joints [oJIA: 20 (47.6%); pJIA: 10 (24.4%)].

The distribution of ultrasound scores (Supplementary Table S3) revealed subtype-specific patterns. In the overall cohort, most joints showed minimal abnormality (GS grade 0–1: 59.6–98.2%), but severe involvement (GS grade 3) was seen at certain sites—the wrist (5.4%), elbow (8.4%), and ankle (6.6%). Compared with oJIA, pJIA patients had a significantly higher prevalence of severe synovitis at the MCP (GS grade 3: 9.5% vs. 0%), PIP (9.8% vs. 0%), and wrist (9.8% vs. 1.2%) joints, with correspondingly higher PD grade 3 signals at the wrist (12.2% vs. 0%) and MCP (7.6% vs. 0%) joints.

Subtype-stratified analysis showed distinct patterns: oJIA had the lowest overall agreement (Kappa = 0.11) but excellent MCP concordance (Kappa = 0.66), whereas pJIA had higher overall agreement (Kappa = 0.15) with optimal wrist concordance (Kappa = 0.60). These subtype-specific differences in joint involvement and clinical–ultrasound concordance support the development of differentiated, subtype-specific standardized ultrasound protocols (Supplementary Figures S1, S2).

### Subtype-specific simplified models

**Figure 1:**
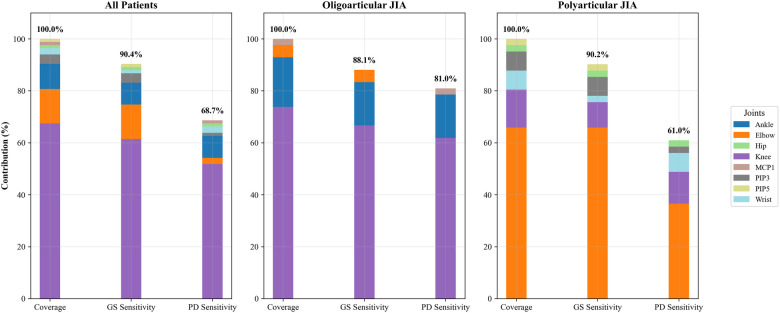
Construction and performance of the subtype-specific simplified ultrasound assessment models. Stacked bar charts show the contribution of each joint to patient coverage, grayscale (GS) sensitivity, and Power Doppler (PD) sensitivity in the different JIA subtypes. Left: all-patient simplified model comprising 8 joint pairs (bilateral elbows, wrists, hips, knees, ankles, MCP1, PIP3, and PIP5; *n* = 83); middle: oligoarticular JIA simplified model comprising 4 joint pairs (bilateral elbows, knees, ankles, and MCP1; *n* = 42); right: polyarticular JIA simplified model comprising 6 joint pairs (bilateral elbows, wrists, hips, knees, PIP3, and PIP5; *n* = 41). Color coding: knee (purple), elbow (orange), ankle (blue), wrist (light blue), hip (green), MCP1 (brown), PIP3 (gray), and PIP5 (light green). In the oligoarticular JIA model, the knee alone accounted for 73% of patient detection, reflecting the predominance of lower-limb large-joint involvement; in the polyarticular JIA model, the elbow contributed most (64%), followed by the wrist (approximately 16%), reflecting the more complex pattern of polyarticular involvement. All models achieved 100% patient coverage, with synovitis defined as a GS score ≥ 2 and/or a PD score ≥ 1.

Using the data-driven stepwise strategy, three simplified models were constructed, each showing clear subtype-specific differences (Figure 1). The oJIA model comprised 4 joint pairs (bilateral elbows, knees, ankles, and MCP1) and achieved 100% patient coverage, with a GS sensitivity of 88.1% and a PD sensitivity of 81.0%. The knee contributed the highest individual yield, with isolated knee involvement detected in 73% of oJIA patients with active disease, reflecting the predominance of lower-limb large-joint involvement in this subtype.

The pJIA model required 6 joint pairs (bilateral elbows, wrists, hips, knees, PIP3, and PIP5) for complete coverage, with a GS sensitivity of 90.2% and a PD sensitivity of 61.0%. The elbow contributed most (approximately 64%), followed by the wrist (approximately 16%), reflecting the widespread polyarticular involvement of pJIA across both large and small joints. The all-patient model comprised 8 joint pairs (bilateral elbows, wrists, hips, knees, ankles, MCP1, PIP3, and PIP5), achieving 100% coverage with a GS sensitivity of 90.4% and a PD sensitivity of 68.7%.

Disease activity stratification showed that PD sensitivity improved markedly in high-disease-activity states across all models: from 78.2% to 92.6% in the all-patient group, from 80.8% to 93.3% in the oJIA group, and from 65.5% to 91.7% in the pJIA group. The pJIA group showed the most pronounced activity-dependent increase in PD sensitivity (26.2 percentage points), reflecting the strong association between Power Doppler signal and active synovial inflammation in polyarticular disease (Supplementary Figure S3).

### Multidimensional performance validation

**Figure 2:**
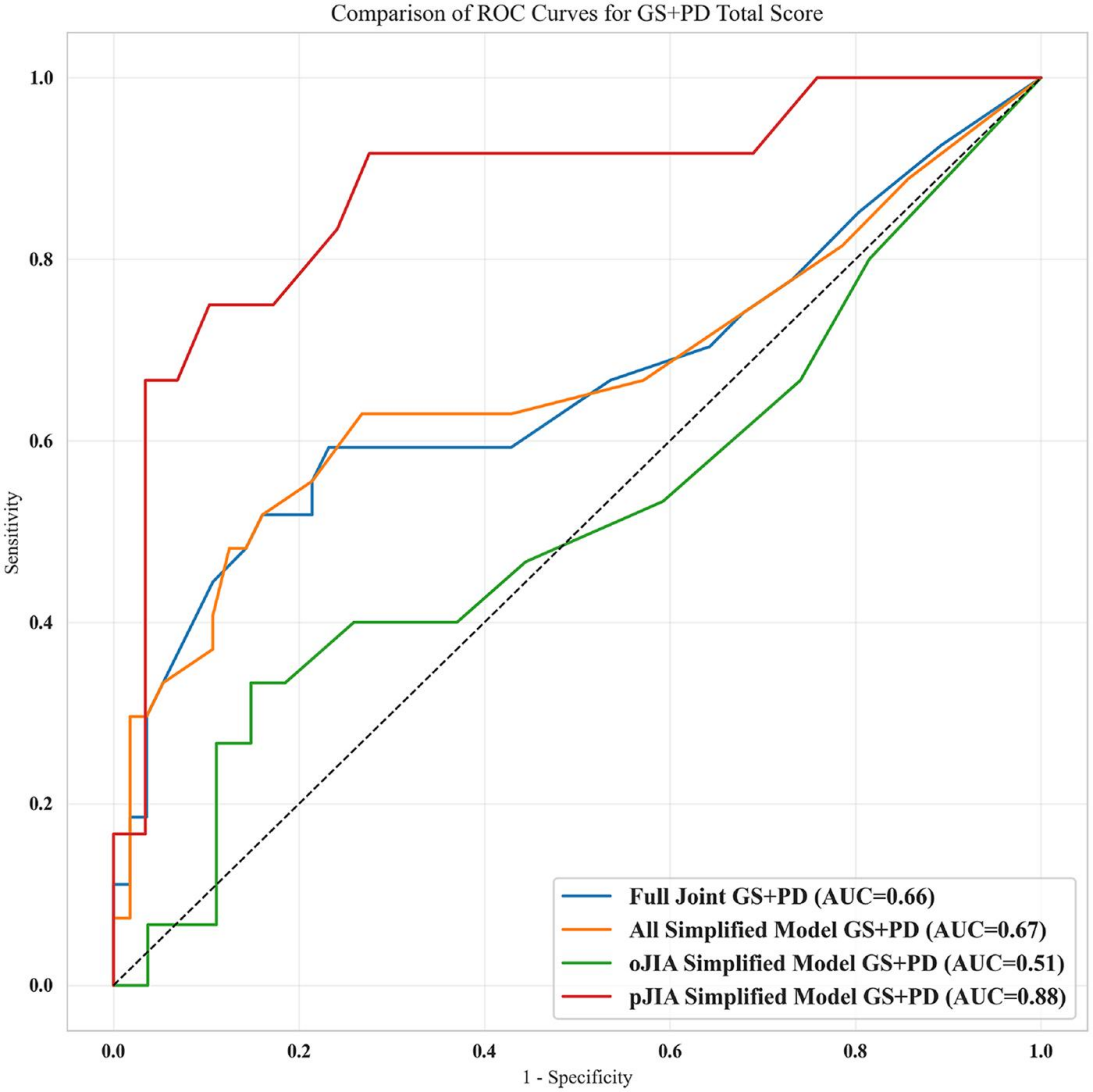
ROC curve comparison of the simplified ultrasound assessment models. Receiver operating characteristic curves compare the diagnostic performance of the different ultrasound assessment strategies for detecting high disease activity (JADAS27 > 4.2 in oligoarticular JIA, or JADAS27 > 8.5 in polyarticular JIA). The models shown are: comprehensive joint GS + PD assessment (blue line, AUC = 0.66), all-patient simplified model GS + PD (orange line, AUC = 0.67), oligoarticular JIA simplified model GS + PD (green line, AUC = 0.51), and polyarticular JIA simplified model GS + PD (red line, AUC = 0.88). The diagonal reference line represents random chance (AUC = 0.50). The polyarticular JIA-specific model demonstrated excellent discriminatory ability.

ROC and forest-plot analyses assessed the ability of the models to discriminate between high and low disease activity. The pJIA model performed best (AUC = 0.88, 95% CI: 0.76–0.95), significantly exceeding the all-patient model (AUC = 0.66, 95% CI: 0.55–0.78) and the oJIA model (AUC = 0.51, 95% CI: 0.35–0.67). At the optimal cutoff of 18.5 points, the pJIA model achieved 85.7% sensitivity and 89.3% specificity (Figure 2). Forest-plot analysis confirmed that the pJIA model had the highest sensitivity for identifying high-disease-activity patients [0.77 (95% CI: 0.48–1.00)], significantly higher than the oJIA [0.34 (95% CI: 0.15–0.56)] and all-patient [0.63 (95% CI: 0.46–0.82)] models.

**Figure 3:**
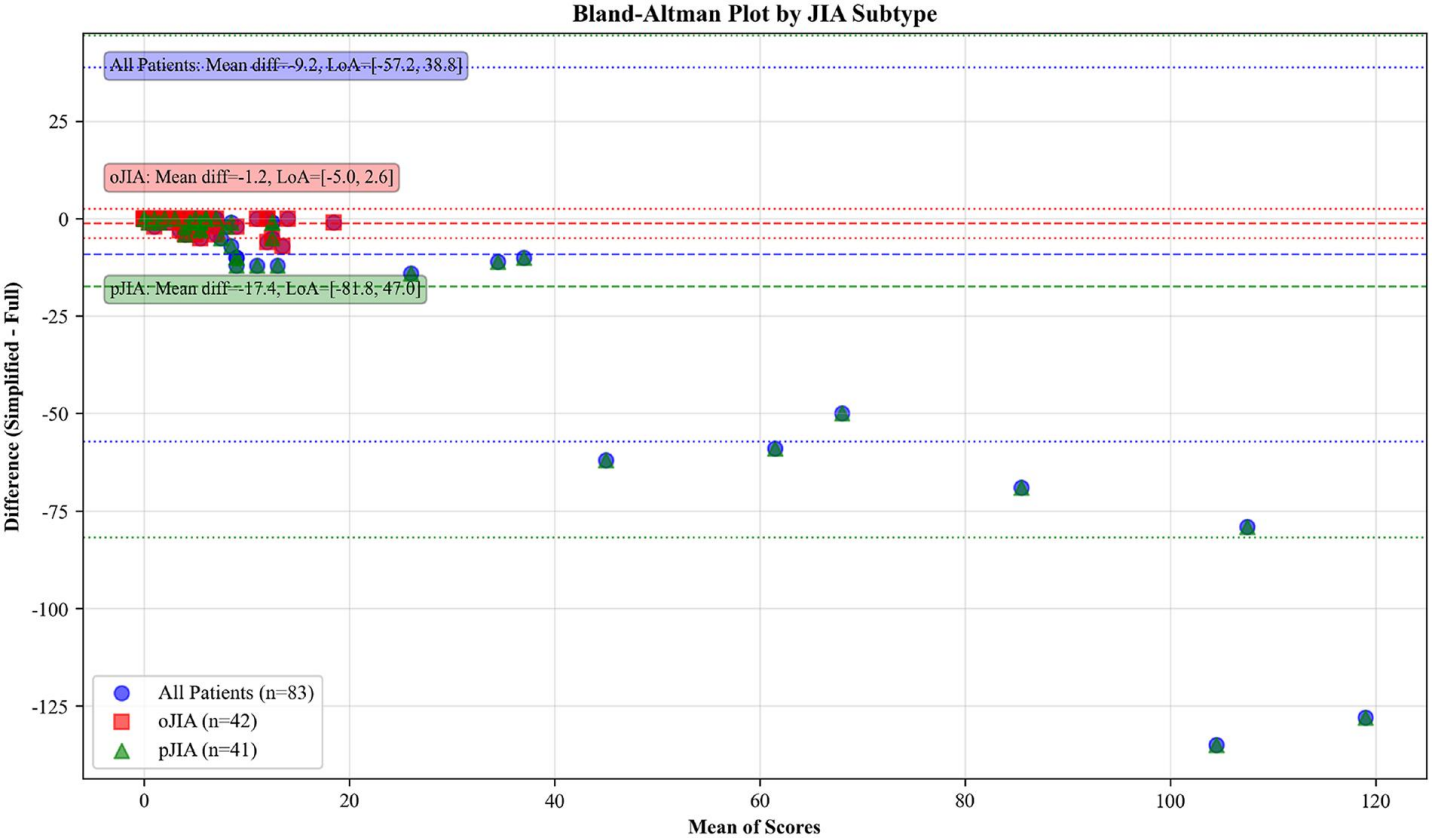
Bland-Altman agreement analysis stratified by JIA subtype. Bland-Altman plots assess the numerical agreement between the simplified and comprehensive joint assessment scores, stratified by JIA subtype. Scatter points represent patients: all patients (blue circles, *n* = 83), oligoarticular JIA (red squares, *n* = 42), and polyarticular JIA (green triangles, *n* = 41). Solid horizontal lines indicate the mean differences, and dashed lines the 95% limits of agreement (LoA). The oligoarticular JIA model showed excellent numerical agreement [mean difference: −1.2; LoA (−5.0, 2.6)], whereas the polyarticular JIA model showed systematic underestimation [mean difference: −17.4; LoA (−81.8, 47.0)]. The all-patient model showed moderate systematic underestimation [mean difference: −9.2; LoA (−57.2, 38.8)].

Bland-Altman analysis revealed distinct roles for the models. The oJIA model showed excellent numerical agreement, with the smallest mean difference (−1.2 points) and narrow 95% limits of agreement [LoA: (−5.0, 2.6) points], establishing its reliability as a long-term monitoring tool. In contrast, the pJIA model showed marked systematic underestimation [mean difference: −17.4 points; 95% LoA (−81.8, 47.0) points], reflecting the challenge of capturing complex polyarticular involvement with a simplified score (Figure 3).

In correlation analysis, the simplified GS + PD score showed a moderate positive correlation with JADAS27 (rs = 0.45, *P* < 0.001), confirming its value in reflecting disease activity. Subtype stratification revealed important differences: the pJIA group showed stronger correlations (JADAS27: rs = 0.68, *P* < 0.001; active joint count: rs = 0.52, *P* < 0.01), whereas the oJIA group showed weaker correlations. Notably, the correlation decreased in high-disease-activity pJIA patients (JADAS27: rs = 0.15–0.19, *P* > 0.05 vs. low activity: rs = 0.43–0.46, *P* < 0.01), suggesting a possible “clinical–imaging dissociation” in which composite clinical scores and local joint inflammation capture different dimensions of disease (Supplementary Figure S4).

Cross-sectional comparison showed that pJIA patients with high disease activity (*n* = 12) had significantly higher simplified GS scores (median: 14.0, IQR: 5.8–21.5) and PD scores (median: 6.0, IQR: 2.0–11.0) than those with low disease activity (*n* = 29; GS median 3.0, IQR: 2.0–5.0; PD median 0, IQR: 0–1.0; both *P* < 0.001; Supplementary Table S2). In contrast, oJIA patients showed no significant difference between high- (*n* = 15) and low- (*n* = 26) activity groups (GS: median 2.0 vs. 2.5, *P* = 0.84; PD: median 0 vs. 0, *P* = 0.22), consistent with the limited discriminatory capacity of the oJIA model (AUC = 0.51).

Decision curve analysis evaluated the practical value of the models in clinical decision-making. Across a threshold-probability range of 0.15–0.65, the pJIA model consistently provided positive net benefit, clearly superior to the “treat-all” and “treat-none” strategies. The oJIA and all-patient models showed more limited net-benefit ranges, further supporting the superiority of the subtype-specific approach (Supplementary Figure S5).

### Treatment-response sensitivity and clinical efficiency

Follow-up analysis of 10 patients with paired baseline and follow-up assessments demonstrated robust treatment-response sensitivity. The all-patient model showed a GS-score SMR of 1.42 and a PD-score SMR of 1.31, both exceeding the high-sensitivity threshold (>0.8). Responsiveness was greater in pJIA (SMR = 1.68); it was lower in oJIA but still met the threshold.

The efficiency gains have clear clinical relevance. Comprehensive 68-joint scanning required 38.8 ± 5.9 min, whereas the simplified models substantially reduced examination time (oJIA, 12.5 ± 4.4 min; pJIA, 13.3 ± 3.2 min; all-patient, 15.3 ± 3.2 min), an approximately 67% reduction. This improvement addresses a key barrier to implementing ultrasound in routine pediatric rheumatology practice while maintaining diagnostic accuracy.

**Figure 4:**
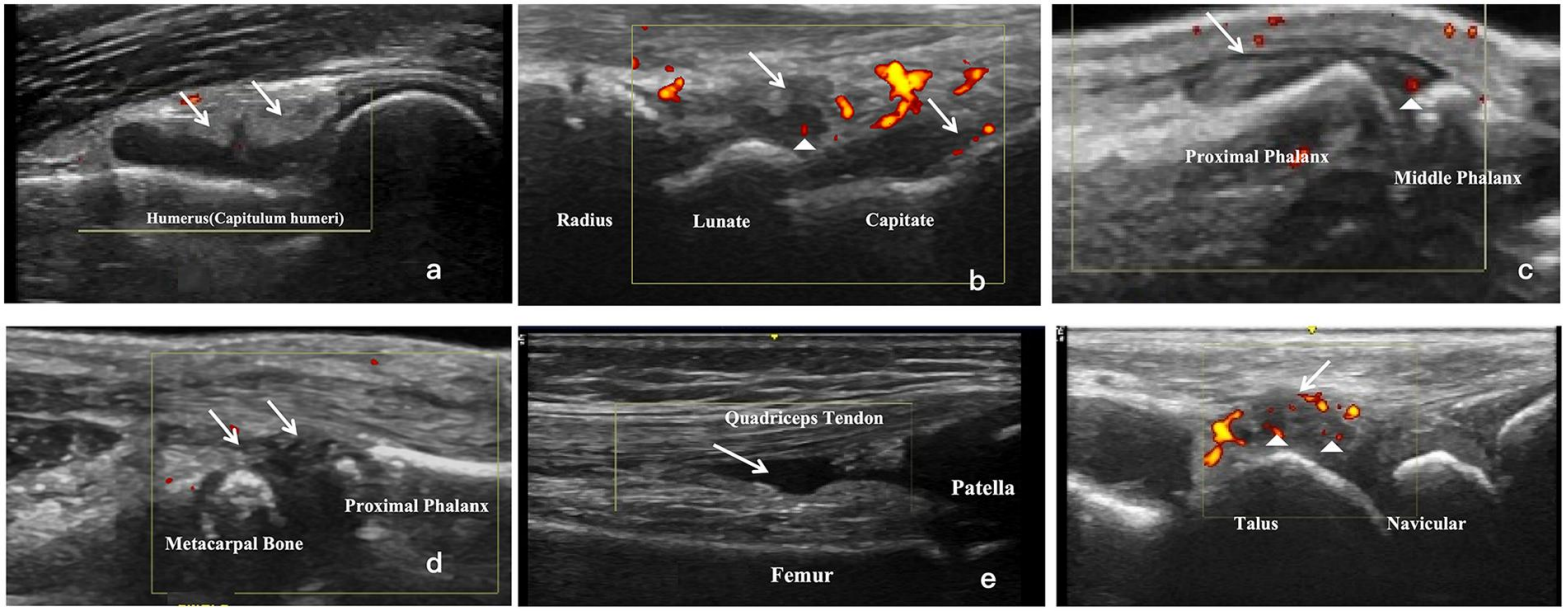
Representative musculoskeletal ultrasound images of key joints included in the subtype-specific simplified assessment models. All images were obtained using high-frequency linear array probes (7–18 MHz) under standardized grayscale (GS) and Power Doppler (PD) settings, in accordance with the pediatric OMERACT scanning protocols. Synovitis severity was graded using a semiquantitative scoring system (GS: 0–3; PD: 0–3). Panels **(a–d)** show joint sites included in the pJIA simplified model, and panels **(e–f)** show joint sites included in the oJIA simplified model. White arrows indicate synovial thickening; arrowheads indicate Power Doppler signal reflecting synovial hypervascularity. All machine-embedded text and institutional identifiers were removed prior to submission. **(a)** Elbow joint, posterior longitudinal scan (pJIA, female, 14 years): marked synovial thickening in the posterior joint recess (arrows), with no detectable Power Doppler signal (GS grade 3, PD grade 0). H, humerus (capitulum humeri). **(b)** Wrist joint, dorsal longitudinal scan over the radiocarpal and midcarpal compartments (pJIA, female, 10 years): dorsal intercarpal synovial thickening (arrows) with active Power Doppler signal indicating synovial hypervascularity (arrowhead; GS grade 2, PD grade 2). R, radius; Lu, lunate; Ca, capitate. **(c)** Third proximal interphalangeal (PIP3) joint, dorsal longitudinal scan (pJIA, male, 9 years): synovial thickening of the dorsal joint recess (arrow) with mild Power Doppler signal (arrowhead; GS grade 3, PD grade 1). PP, proximal phalanx; MP, middle phalanx. **(d)** First metacarpophalangeal (MCP1) joint, dorsal longitudinal scan (oJIA, male, 3 years): marked synovial thickening occupying the dorsal recess (arrows), with no detectable Power Doppler signal (GS grade 3, PD grade 0). MC, metacarpal bone; PP, proximal phalanx. **(e)** Knee joint, suprapatellar longitudinal scan (oJIA, male, 7 years): synovial thickening and joint effusion within the suprapatellar recess (arrow), with no detectable Power Doppler signal (GS grade 2, PD grade 0). F, femur; QT, quadriceps tendon; P, patella. **(f)** Ankle joint, anterior longitudinal scan over the tibiotalar joint (oJIA, male, 7 years): synovial thickening (arrow) with prominent Power Doppler signal (arrowheads; GS grade 3, PD grade 2). Ta, talus; Na, navicular. GS, grayscale; PD, Power Doppler; pJIA, polyarticular juvenile idiopathic arthritis; oJIA, oligoarticular juvenile idiopathic arthritis; PIP, proximal interphalangeal; MCP, metacarpophalangeal.

Representative grayscale and Power Doppler images of the key joints in the simplified models are shown in Figure 4, illustrating the characteristic sonographic features of synovitis at pJIA-related joint sites (elbow, wrist, PIP3; panels a–c) and oJIA-related joint sites (MCP1, knee, ankle; panels d–f), including marked synovial thickening and prominent Power Doppler signal.

## Discussion

Musculoskeletal ultrasound is a non-invasive, radiation-free, and increasingly accessible bedside imaging modality in pediatric rheumatology that enables real-time assessment of synovial inflammation. In this prospective study, we developed subtype-specific simplified ultrasound assessment systems for the two most common JIA subtypes, oJIA and pJIA, providing clinicians with practical, evidence-based tools for individualized disease management. For pJIA, we recommend the 6-joint-pair model (bilateral elbows, wrists, hips, knees, PIP3, and PIP5) for routine disease activity assessment and treatment decision-making; this model showed excellent discrimination of high disease activity (AUC = 0.88, 95% CI: 0.76–0.95). In cross-sectional analysis, high-activity pJIA patients had significantly higher simplified GS scores (median: 14.0, IQR: 5.8–21.5; *n* = 12) and PD scores (median 6.0, IQR: 2.0–11.0) than low-activity patients (GS median 3.0, IQR: 2.0–5.0; PD median 0, IQR: 0–1.0; *n* = 29; both *P* < 0.001). For oJIA, we recommend the 4-joint-pair model (bilateral elbows, knees, ankles, and MCP1), primarily for longitudinal monitoring and treatment-response assessment, given its excellent numerical agreement with comprehensive assessment [mean difference: −1.2; 95% LoA (−5.0, 2.6)] rather than its discrimination of disease activity (AUC = 0.51). Both models reduced examination time by approximately 67% (from 38.8 ± 5.9 to 12.5–13.3 min) while maintaining 100% patient coverage, directly addressing the principal barrier to routine ultrasound use in pediatric rheumatology. Collectively, these findings give pediatric rheumatologists a practical, subtype-tailored framework that can be readily integrated into routine workflows to support objective, efficient, and individualized disease assessment.

The differing performance of the two models reflects the distinct pathobiology of the two subtypes. pJIA is an antigen-driven, lymphocyte-mediated autoimmune disease characterized by activation of T-helper type 1 (Th1) and type 17 (Th17) cells, which produce pro-inflammatory cytokines such as interferon-*γ*, interleukin-17, and tumor necrosis factor-α, together with impaired regulatory T-cell function and loss of immune tolerance (16–18). This widespread inflammation manifests on ultrasound as more pronounced synovial thickening and joint effusion, with increased PD signal reflecting active synovial neovascularization that correlates with disease activity in JIA (6, 11).

From a histopathological standpoint, the vascular signals detected by Power Doppler correspond specifically to neovascularization within the subintimal layer of the synovial membrane, not the intimal (lining) layer, which is physiologically avascular and penetrated only by nerve fibers. In chronic inflammation, the intimal layer undergoes synoviocyte hyperplasia or, in fibrotic synovium, progressive “delamination” with denudation of the lining surface; in contrast, the subintimal layer shows capillary hyperplasia, telangiectasia, and extensive microvascular remodeling, including arteriovenous shunt-like elements, which constitute the histological substrate of the Power Doppler signal. Although the two layers cannot be distinguished on ultrasound, this sono-histological correspondence provides a firm pathophysiological rationale for using Power Doppler signal intensity as a surrogate marker of active synovial inflammation (26). This capacity to detect inflammatory vascularity enables the 6-joint-pair pJIA model to capture complex polyarticular involvement and achieve excellent diagnostic performance (AUC = 0.88), and decision curve analysis confirmed positive net benefit across the critical threshold range (0.15–0.65), providing a reliable tool for identifying high-risk patients who require intensified treatment.

In contrast, oJIA typically involves more limited immune activation, predominantly affecting the large joints of the lower limbs, with milder inflammation and simpler pathology (27, 28). The limited discrimination of the oJIA model (AUC = 0.51) is not a defect but reflects the characteristically low disease activity and simple pathology of these patients. Here, the principal value of ultrasound lies not in distinguishing high from low disease activity but in providing reliable, reproducible longitudinal monitoring, which is well suited to the generally favorable prognosis of most oJIA patients (18).

An unexpected finding merits comment: oJIA showed lower clinical–ultrasound agreement (κ = 0.11) than pJIA (κ = 0.15), despite its characteristic large-joint predominance. Subclinical synovitis was common in oJIA, reflecting the combined effects of anatomical constraints and subtle inflammatory signals. The literature confirms that small joints are the most frequent sites of missed subclinical synovitis (20), with 51.5% of ultrasound-positive joints being clinically silent in JIA, particularly the wrists, PIP joints, and feet (9); recent multicenter data report subclinical synovitis in 30% of joints, more often in low-disease-activity states (median JADAS ≈ 4.8) (7). In oJIA, low inflammatory activity (median JADAS-27: 4.0) produces minimal clinical signs even in large joints (5), and when small joints are involved, anatomical constraints compound the difficulty (wrist κ = −0.04; PIP κ = 0.08). In our cohort, pJIA showed relatively better small-joint concordance (wrist κ = 0.60), probably because these joints exhibited pronounced inflammation that facilitated clinical detection despite anatomical challenges. These findings underscore the complementary value of ultrasound: in oligoarticular disease, for detecting subtle inflammation regardless of joint size; and in polyarticular disease, for identifying residual subclinical synovitis.

The reduced correlation in high-activity pJIA patients (JADAS27 correlation falling from 0.43–0.46 to 0.15–0.19) may reflect “clinical–imaging dissociation”. JADAS27 integrates multidimensional information—patient-reported outcomes, physician assessment, and inflammatory markers—and mainly reflects systemic disease burden (22), whereas ultrasound primarily captures local structural change and vascularity. In high-activity states, some patients present predominantly with systemic inflammation, pain sensitization, or extra-articular features while local joint inflammation remains relatively stable, so the two methods capture different dimensions of disease (7). This phenomenon highlights the need for multidimensional assessment in complex disease states. Importantly, despite the weaker correlation, the pJIA model retained excellent diagnostic performance (AUC = 0.88), indicating that ultrasound has independent clinical value and should complement, rather than replace, conventional clinical indicators.

This study also found that PD sensitivity improved significantly in high-disease-activity states (oJIA: 80.8% to 93.3%; pJIA: 65.5% to 91.7%), consistent with the role of PD signal in reflecting angiogenesis and inflammatory activity. Recent studies confirm that standardized PD signals correlate significantly with B-mode synovitis severity (29), supporting PD as an objective marker of disease activity (30, 31). Nevertheless, PD findings require careful interpretation, as the grading of mild signals is partly subjective and studies disagree on their predictive value: Magni-Manzoni et al. (12) reported limited value for predicting flares, and De Lucia et al. (10) found inconsistent associations between PD findings and clinical outcomes. Despite these caveats, the value of PD in identifying subclinical inflammation and predicting relapse has been confirmed by multiple studies, providing important imaging evidence for the precision management of JIA (7, 8).

## Study limitations and future directions

This study has several limitations. First, the single-center design and moderate sample size (*n* = 83) limit the external validity of the proposed models; although the sample met the pre-specified power requirements, the high-activity pJIA subgroup (*n* = 12) may limit the precision of the AUC estimate, and the follow-up cohort (*n* = 10) is insufficient for formal active-to-remission paired analysis. Multicenter prospective validation in larger, more diverse JIA populations is therefore needed. Second, only oJIA and pJIA were included; subgroup analyses by ANA/RF status or by persistent vs. extended oligoarticular classification were precluded by sample size, although RF-positive pJIA patients (*n* = 8) showed characteristic MCP2/3 involvement (62.5% each), warranting RF-specific model development in future studies. Third, we used conventional Power Doppler with validated pediatric-specific protocols (7); whether newer modalities—such as the PIUS protocol (32, 33) or high-sensitivity techniques (SMI/MVI)—would enhance performance remains to be determined. Fourth, the absence of comparator groups (sJIA, infectious arthritis, healthy controls) precludes assessment of differential diagnostic specificity, which will require dedicated multicenter case-control studies.

Future research should include: (1) multicenter prospective validation across diverse JIA subtypes; (2) evaluation of newer ultrasound techniques (PIUS protocols and high-sensitivity Doppler) to optimize diagnostic accuracy; (3) integration of artificial intelligence to improve standardization (34, 35); and (4) development of multimodal models combining ultrasound with molecular biomarkers for JIA precision medicine.

## Conclusions

The subtype-specific simplified ultrasound assessment systems developed in this study substantially improved examination efficiency while maintaining high diagnostic accuracy, representing an important methodological advance for pediatric rheumatology. By addressing the practical limitations of comprehensive assessment, this approach lays a foundation for the individualized management of JIA and supports the broader movement of pediatric rheumatology toward precision medicine.

## Data Availability

The datasets presented in this study can be found in online repositories. The names of the repository/repositories and accession number(s) can be found in the article/Supplementary Material.
