# Supplementary material for: Development and validation of subtype-specific simplified ultrasound assessment systems for juvenile idiopathic arthritis: a prospective observational study
Source: Front Pediatr. 2026 Jul 6;14:1876983. doi: 10.3389/fped.2026.1876983 (PMC13381455; doi:10.3389/fped.2026.1876983)
Supplement: Supplementary file 1 [file Datasheet1.docx]

**Supplementary Material 1**

**Comprehensive scanning protocols and joint-specific scoring criteria for 68-joint ultrasound assessment**

| **Joint** | **Scan Plane/Position** | **Scanning Method** | **GS Grading Criteria** |
| --- | --- | --- | --- |
| **SHOULDER** | Biceps tendon groove - transverse | Patient seated, forearm flexed on thigh, probe transverse over biceps groove | 0: No tenosynovitis 1: Tenosynovitis present |
|  | Posterior transverse (internal/external rotation) | Probe posterior transverse, assess glenohumeral posterior recess | 0: No effusion  1: Effusion visible only in external rotation  2: Effusion in both internal and external rotation  3: Complete capsular distension |
| **ELBOW** | Anterior (humero-ulnar/humero-radial) | Forearm extended palm up at sides, sagittal plane | 0: Recess not expanded  1: <50% below α-line  2: >50% below α-line  3: Beyond α-line |
|  | Posterior (olecranon fossa) | Elbow flexed 90°, sagittal plane | 0: Empty recess  1: <25% filling  2: 25-50% filling  3: >50% filling |
| **WRIST** | Dorsal transverse | Transverse scan over Lister's tubercle region | 0: No tenosynovitis  1: Tenosynovitis present |
|  | Dorsal transverse (radioulnar) | Transverse scan proximal wrist showing radius/ulna. Define Q-point (apex, 90°) and R-point (mid-arc, 45°) on ulnar dome surface | 0: No synovial recess expansion  1: Synovitis along ulna not exceeding R-point  2: Exceeding R-point but not Q-point  3: Exceeding Q-point |
|  | Dorsal longitudinal axis | Palm down, scan radial/ulnar wrist (radial: radiocarpal, lunate, capitate, metacarpal; ulnar: triquetrum, hamate) | 0: No synovial distension  1: <50% of β-line  2: ≥50% of β-line  3: Beyond β-line |
| **HAND** | MCP/PIP/DIP - dorsal longitudinal | Palm down, sagittal scan of joint midline | 0: Limited to lower 1/3 of fat pad  1: Not exceeding γ-line  2: Exceeding γ-line, proximal synovium parallel to metacarpal shaft, wedge-shaped  3: Exceeding γ-line and elevating tendon, rounded proximally |
|  | MCP/PIP/DIP - volar longitudinal | Palm up, sagittal scan of joint midline | 0: Synovium not visible or only narrow hypoechoic band  1: Thin parallel structure  2: Proximal rounded/cystic not exceeding metacarpal head  3: Exceeding metacarpal head and elevating volar plate |
|  | Tendon sheaths (longitudinal) | Palm up, sagittal scan of joint midline | 0: No tenosynovitis 1: Tenosynovitis present |
| **HIP** | Anterior sagittal scan parallel to femoral neck | Supine with hip neutral and 15° external rotation | 0: Capsule parallel to femoral head/neck; bilateral difference <2.0 mm；  1: Capsular distension with concave shape to femoral neck；  2: Capsular distension with straight shape to femoral neck；  3: Capsular distension with convex shape to femoral neck |
| **KNEE** | Suprapatellar recess sagittal | Supine with 30° knee flexion, sagittal scan above patella | 0: No synovitis, normal joint space or physiological slight widening  1: <50% filling below quadriceps tendon  2: >50% filling below quadriceps tendon  3: Significant capsular distension |
|  | Medial/lateral parapatellar | Transverse view at mid-patella (or maximum distension), showing patella | 0: No or narrow effusion/synovial thickening in parapatellar depression  1: <1/3 joint capsule filling  2: 1/3 to 2/3 capsule filling  3: >2/3 capsule filling |
| **ANKLE** | Tibiotalar (medial sagittal) | Foot flat, sagittal scan of anterior recess showing medial distal tibia and talus | 0: No effusion  1:Mild concave effusion/thickening (<25% filling)  2:Moderate convex effusion/thickening (25-50%)  3: Convex filling >50% |
|  | Talonavicular | Midline longitudinal: proximal at talus, distal to navicular | 0: Normal joint space, angular/V-shaped  1: Widening from angular to mild distension  2:Convex expansion ≤50% of visible bony landmarks  3:Convex expansion >50% of landmarks |
|  | Subtalar - anterior | Medial: probe anterior to medial malleolus, showing talus-subtalar support band | Same as talonavicular |
|  | Subtalar - posterior | Lateral: probe perpendicular to plantar surface along calcaneal sinus, showing talus-calcaneus | Same as talonavicular |
|  | Tendon sheaths (transverse) | Medial flexor tendons: posterior to medial malleolus<br>Lateral tendons: posterior to lateral malleolus | 0: No tenosynovitis  1: Tenosynovitis present |
| **FOOT** | MTP/PTP/DTP - dorsal longitudinal | Similar to finger MCP scanning method | 0: Effusion/thickening limited to lower 1/3 of joint depression  Grades 1-3: Same as finger MCP criteria |
|  |  |  |  |

**Anatomical Reference Lines:**

- **α-line:** Elbow anterior recess reference line (connecting proximal radial fossa and capitellum edge)
- **β-line:** Wrist dorsal reference line (connecting radial head to capitate)
- **γ-line:** Line extending from most superficial point of metacarpal head to proximal phalanx base

**General Scoring Principles:**

- When scoring is ambiguous, assign the lower grade
- Distinguish physiological fluid from pathological synovitis
- PD scoring excludes growth-related nutrient vessels and artifacts; only intra-synovial signal is assessed
- **PD grading:** 0 = no signal (excluding nutrient vessels); 1 = 1-3 signals; 2 = >3 signals or <50% confluence; 3 = >50% confluent signal
