# Supplementary material for: Development and validation of subtype-specific simplified ultrasound assessment systems for juvenile idiopathic arthritis: a prospective observational study
Source: Front Pediatr. 2026 Jul 6;14:1876983. doi: 10.3389/fped.2026.1876983 (PMC13381455; doi:10.3389/fped.2026.1876983)
Supplement: Supplementary file 2 [file Datasheet2.docx]

Supplementary Material 2

**Ultrasound grading atlas showing grayscale and power Doppler scoring (Grades 0-3) across multiple joint sites, stratified by pediatric age groups (Figures SB1-SB21).**

| 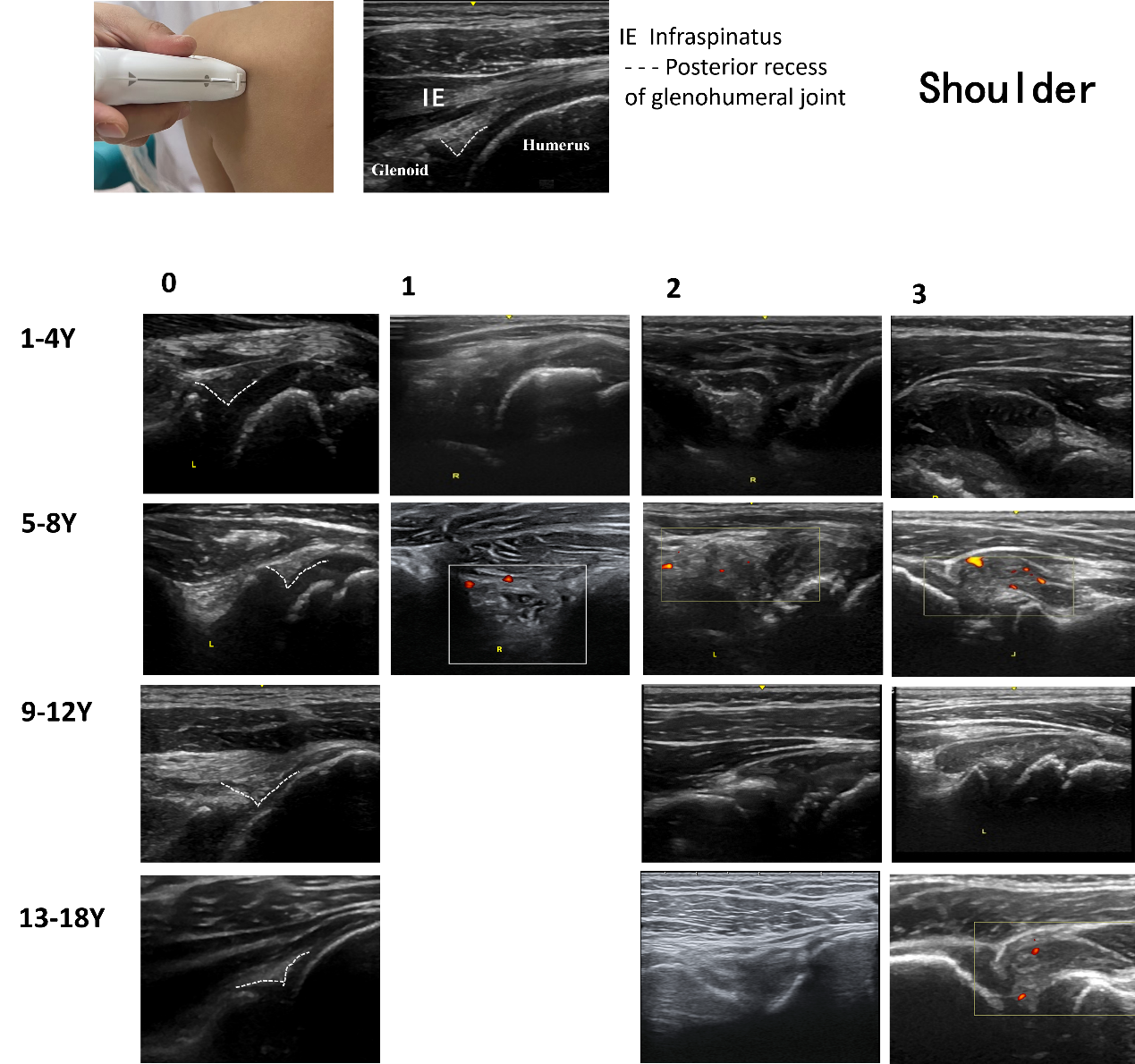 |
| --- |
| Figure SB1. Ultrasound grading criteria for shoulder joint and examples in different age groups  Scanning method: Probe placed posteriorly in transverse position to evaluate posterior recess of glenohumeral joint.  Grading criteria: Grade 0: No effusion; Grade 1: Effusion visible only in external rotation; Grade 2: Effusion visible in both internal and external rotation; Grade 3: Complete distension of joint capsule. |
| 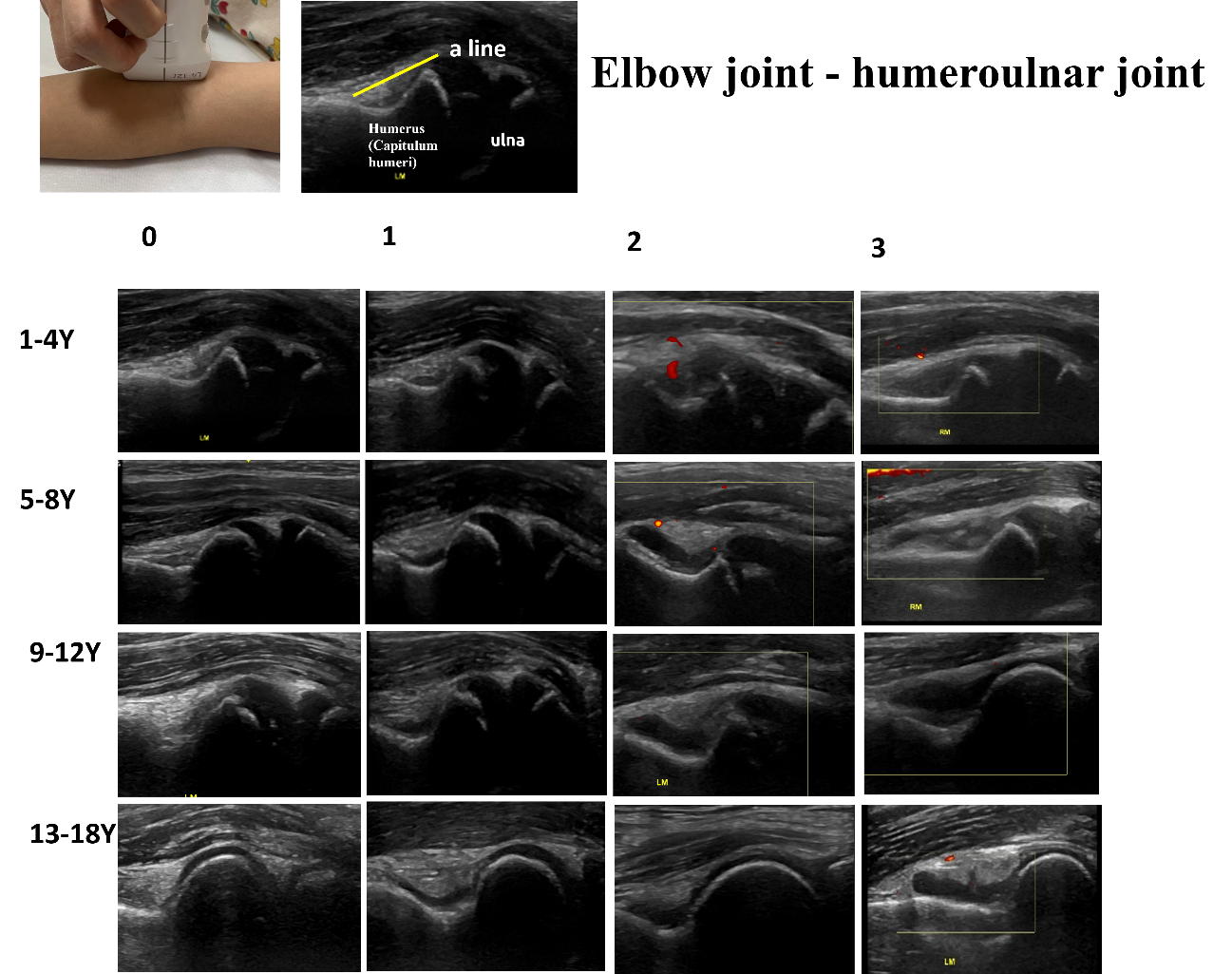 |
| Figure SB2. Ultrasound grading criteria for anterior elbow joint  Scanning method: Forearm extended with palm up, positioned at side of body, sagittal scanning of Ulnohumeral joint.  Grading criteria: Grade 0: No recess expansion; Grade 1: <50% below α-line; Grade 2: >50% below α-line; Grade 3: Beyond α-line. |
| 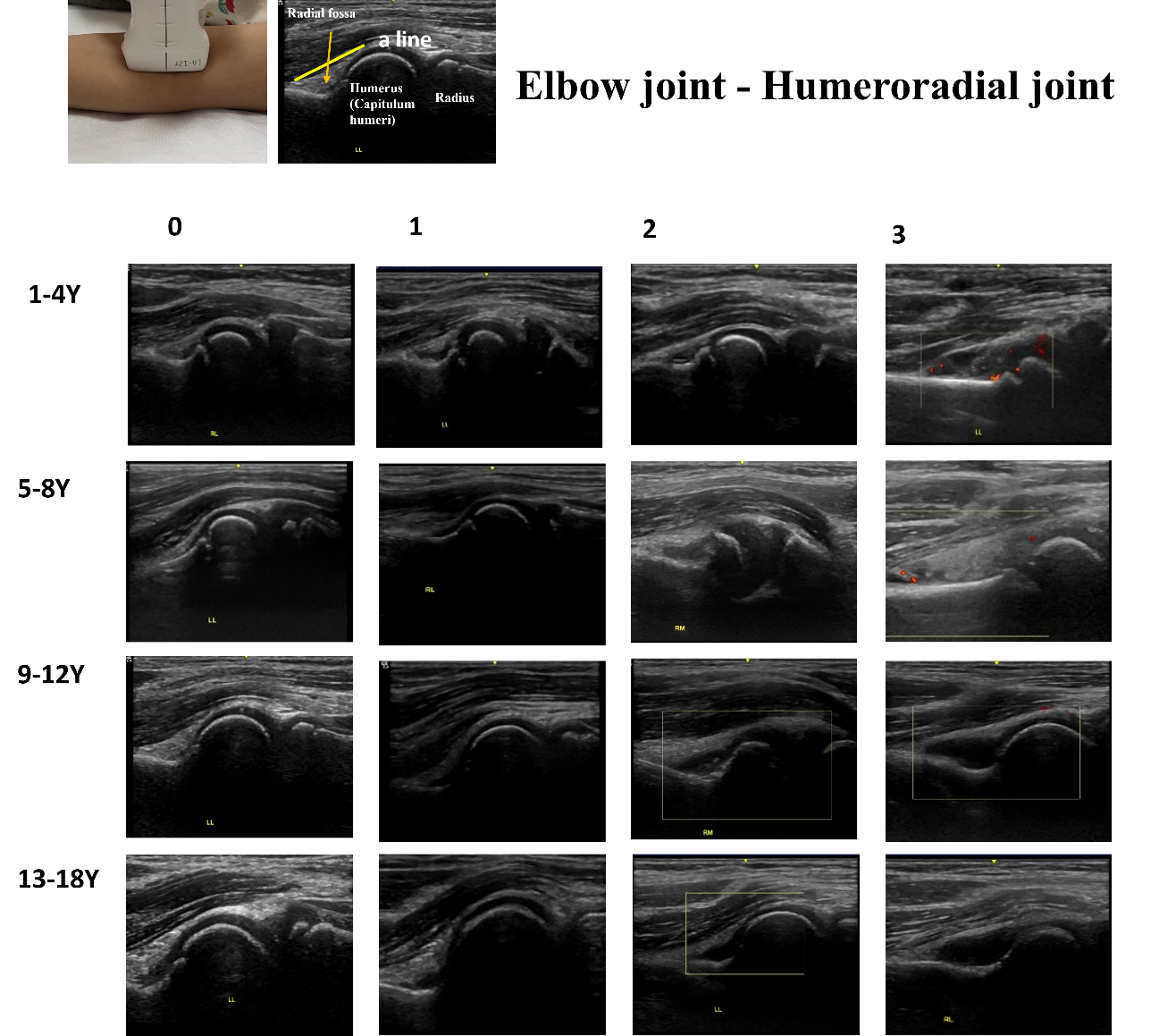 |
| Figure SB3. Ultrasound grading criteria for anterior elbow joint  Scanning method: Forearm extended with palm up, positioned at side of body, sagittal scanning of radio-humeral joint.  Grading criteria: Grade 0: No recess expansion; Grade 1: <50% below α-line; Grade 2: >50% below α-line; Grade 3: Beyond α-line. |
| 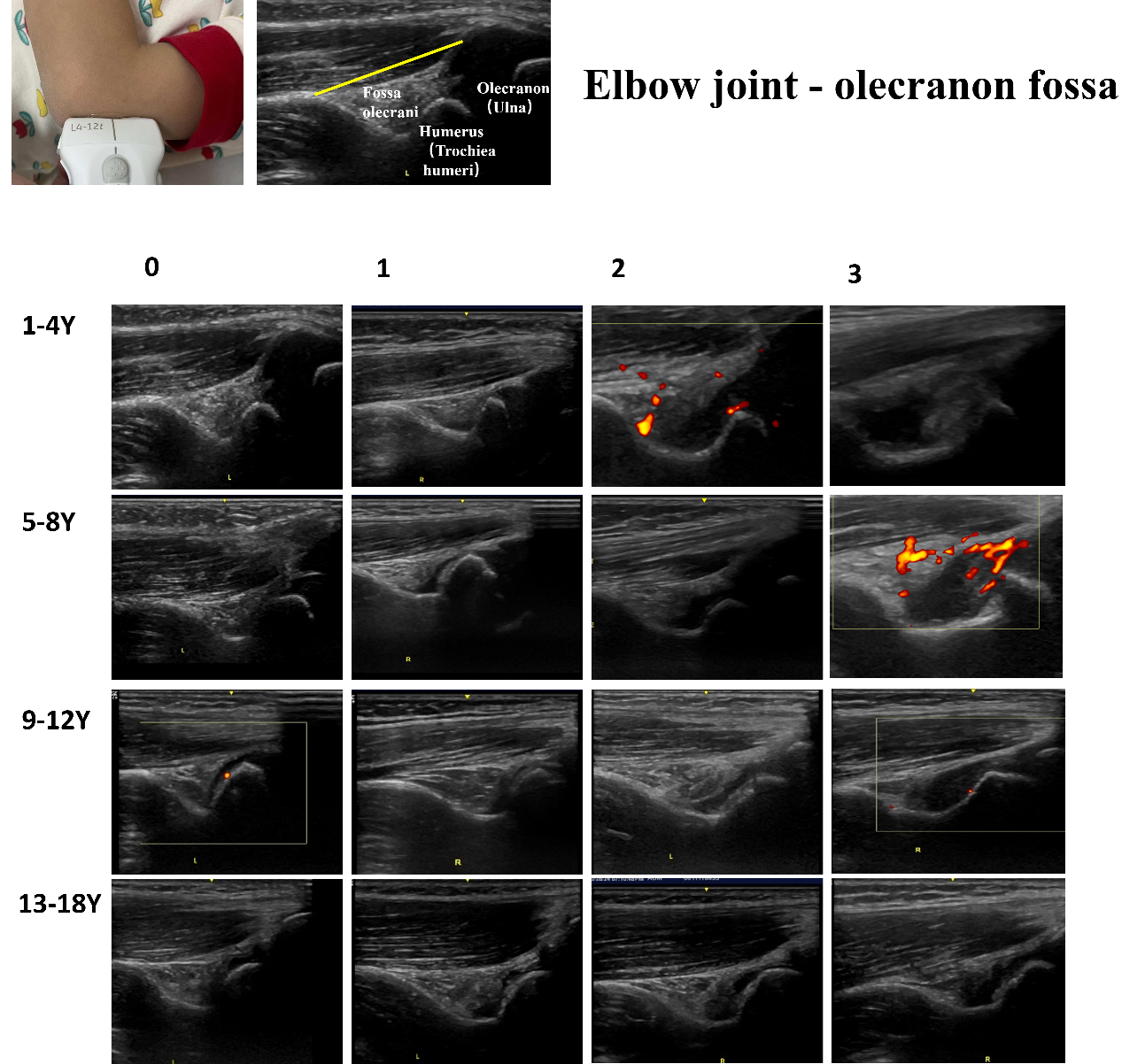 |
| Figure SB4. Ultrasound grading criteria for olecranon fossa  Scanning method: Elbow flexed 90°, sagittal scanning of olecranon fossa  Grading criteria: Grade 0: Empty recess; Grade 1: <25% filling; Grade 2: 25-50% filling; Grade 3: >50% filling |
| 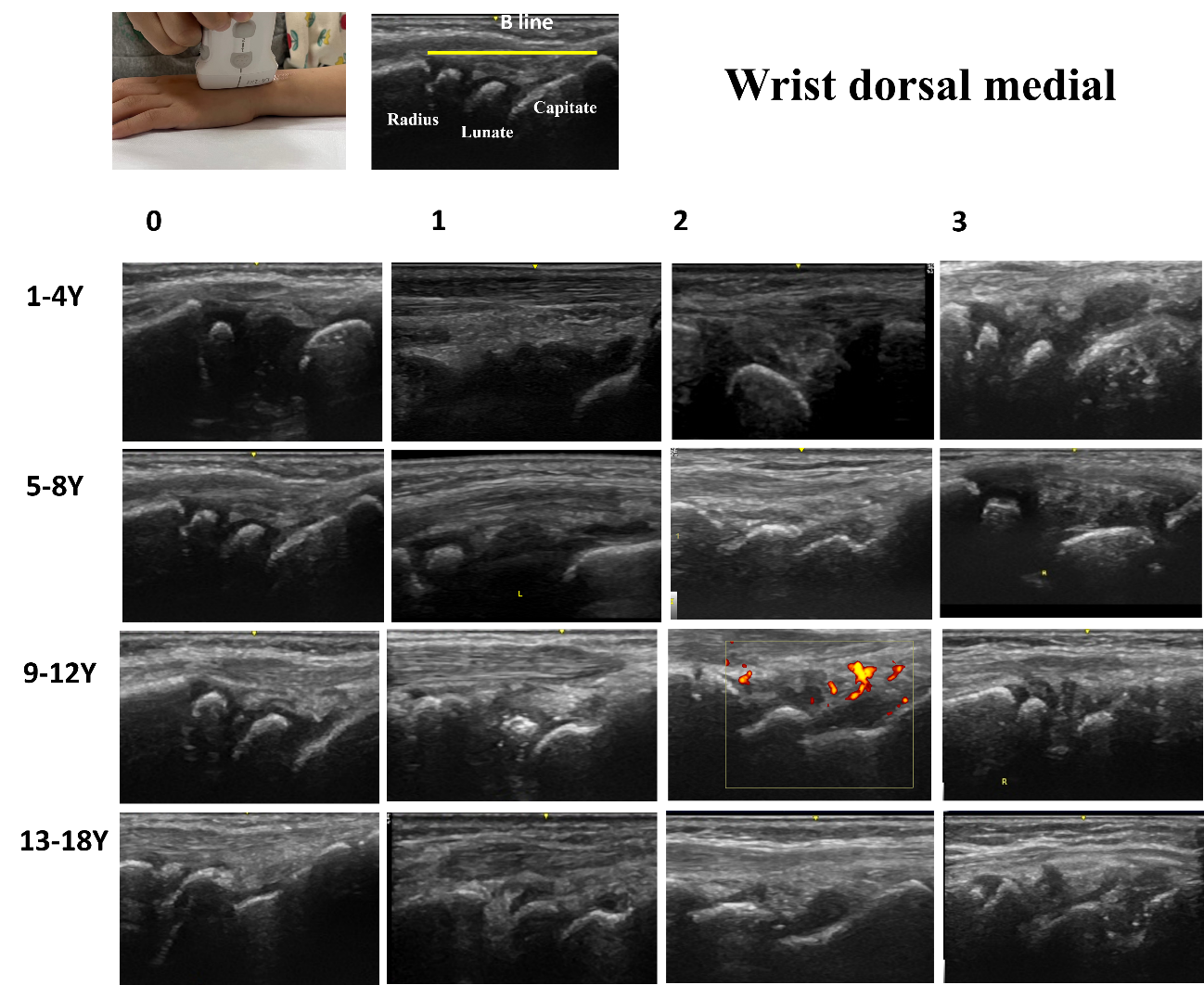 |
| Figure SB5. Ultrasound grading criteria for intercarpal joints  Scanning method: Palm down, scanning radial and ulnar aspects of wrist (radial side showing radiocarpal, lunate, capitate, metacarpal; ulnar side showing triquetrum and hamate)  Grading criteria: Grade 0: No synovial distension; Grade 1: <50% of β-line; Grade 2: ≥50% of β-line; Grade 3: Beyond β-line |
| 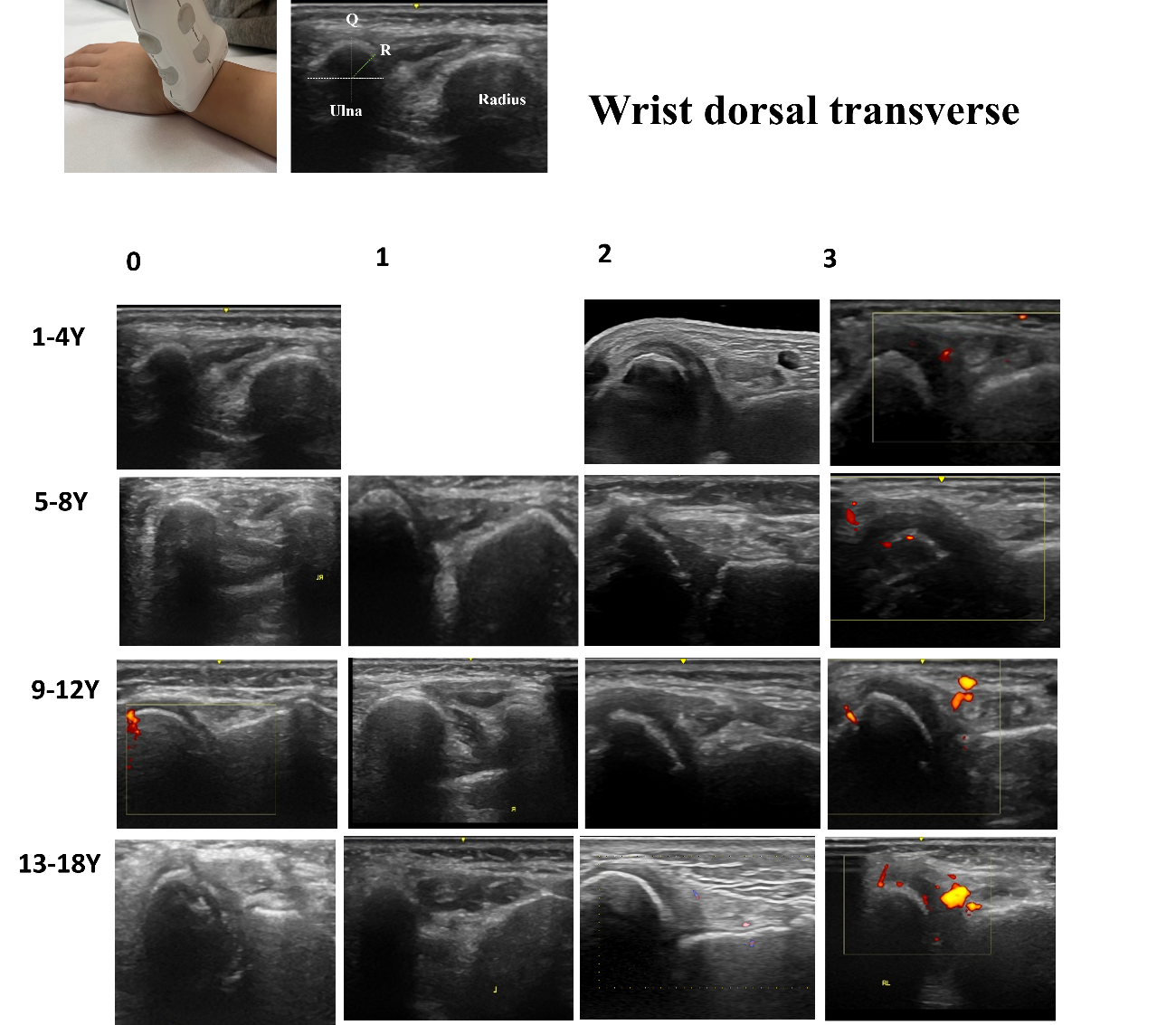 |
| Figure SB6. Ultrasound grading criteria for distal radioulnar joint  Scanning method: Transverse scanning of proximal wrist showing radius and ulna, defining Q-point (apex at 90°) and R-point (mid-arc at 45°) on ulnar dome  Grading criteria: Grade 0: No synovial recess expansion; Grade 1: Synovitis not exceeding R-point; Grade 2: Synovitis exceeding R-point but not Q-point; Grade 3: Synovitis exceeding Q-point |
| 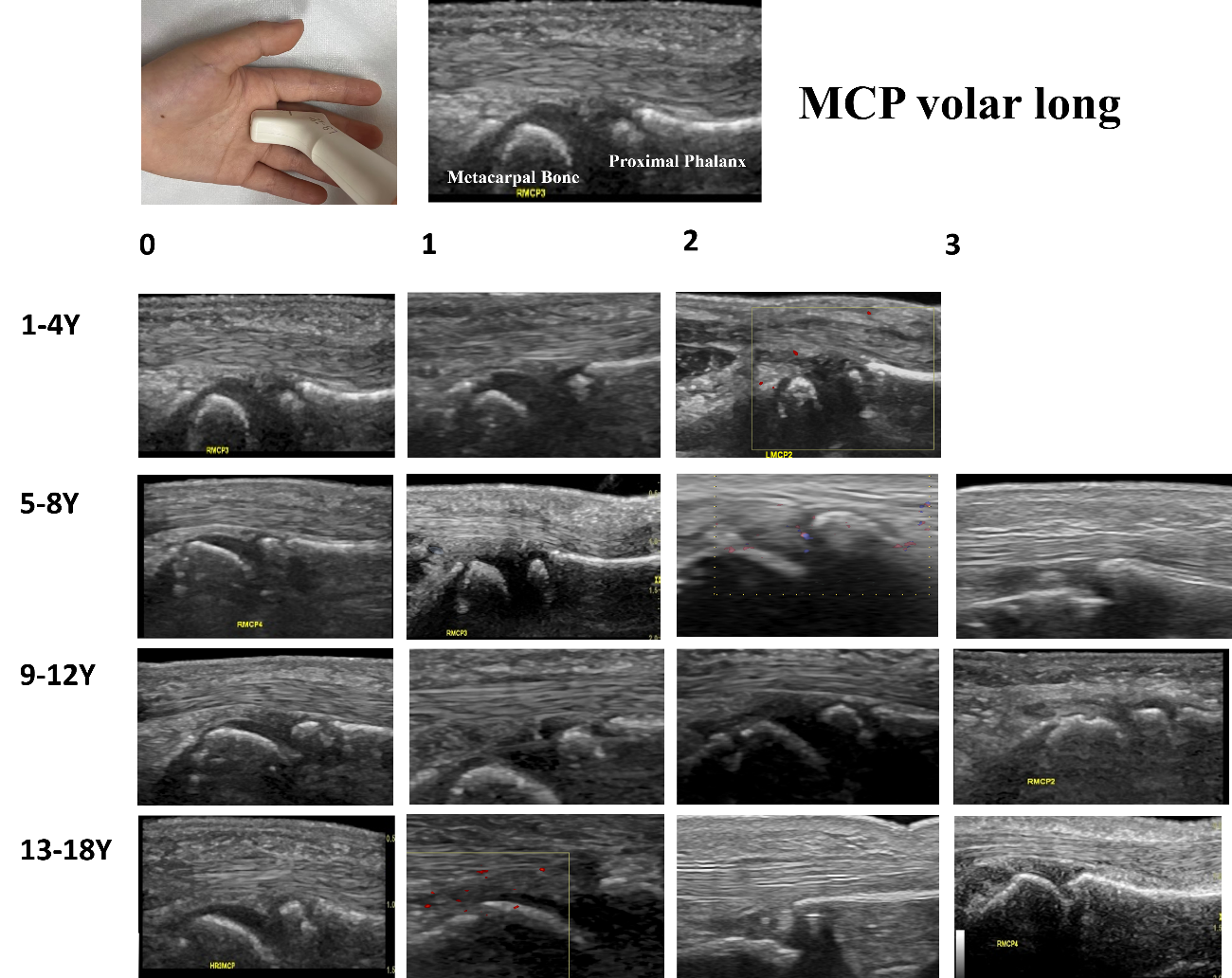 |
| Figure SB7. Ultrasound grading criteria for volar longitudinal MCPjoints  Scanning method: Palm up, sagittal scanning of joint midline.  Grading criteria: Grade 0: Synovium not visible or only narrow hypoechoic band; Grade 1: Thin parallel structure; Grade 2: Proximal rounded/cystic not exceeding metacarpal head; Grade 3: Exceeding metacarpal head and elevating volar plate. |
| 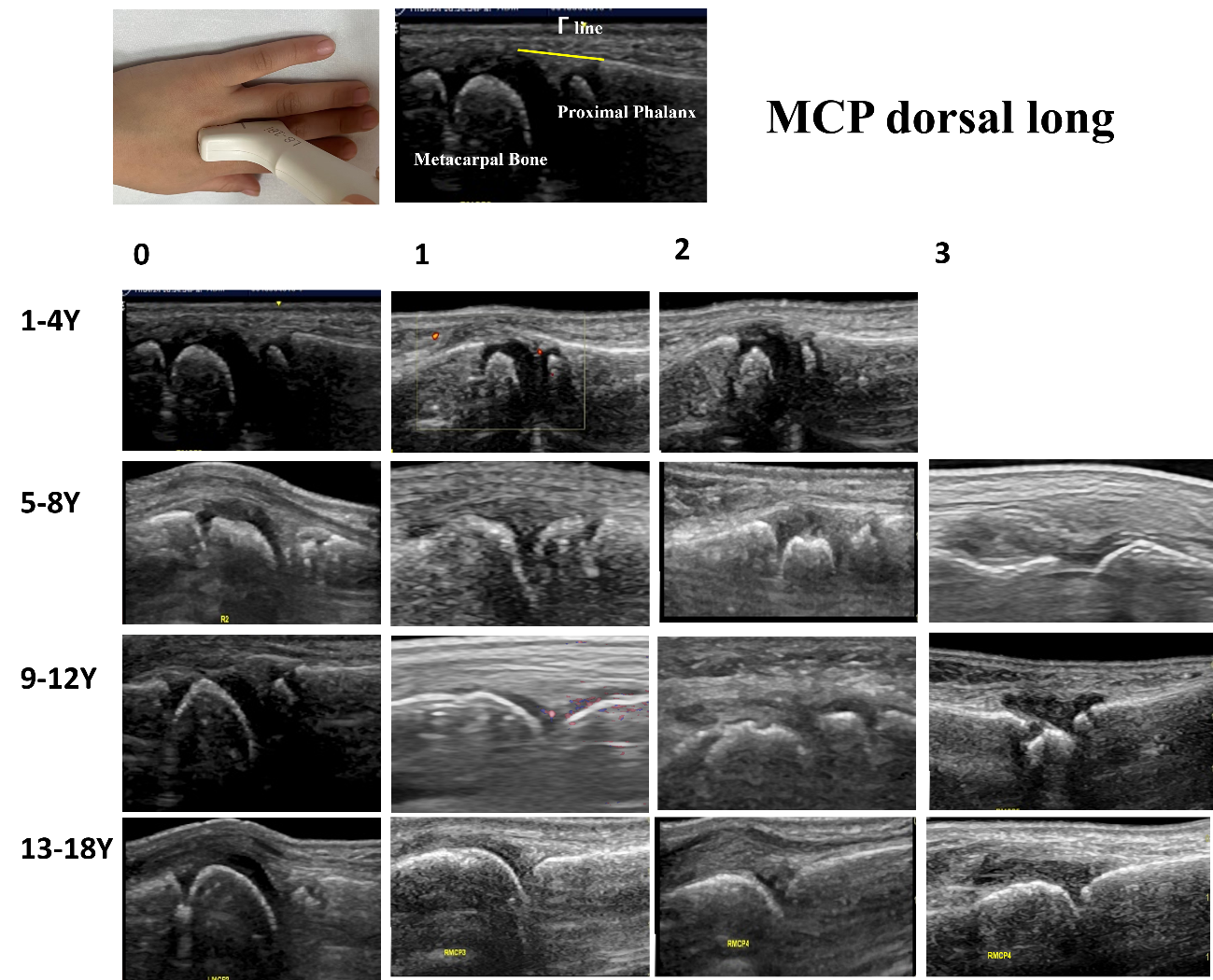 |
| Figure SB8. Ultrasound grading criteria for dorsal longitudinal MCP joints  Scanning method: Palm down, sagittal scanning of joint midline.  Grading criteria: Grade 0: Limited to lower 1/3 of fat pad; Grade 1: Not exceeding γ-line; Grade 2: Exceeding γ-line with proximal synovium parallel to metacarpal shaft, wedge-shaped; Grade 3: Exceeding γ-line and elevating tendon, rounded proximally. |
| 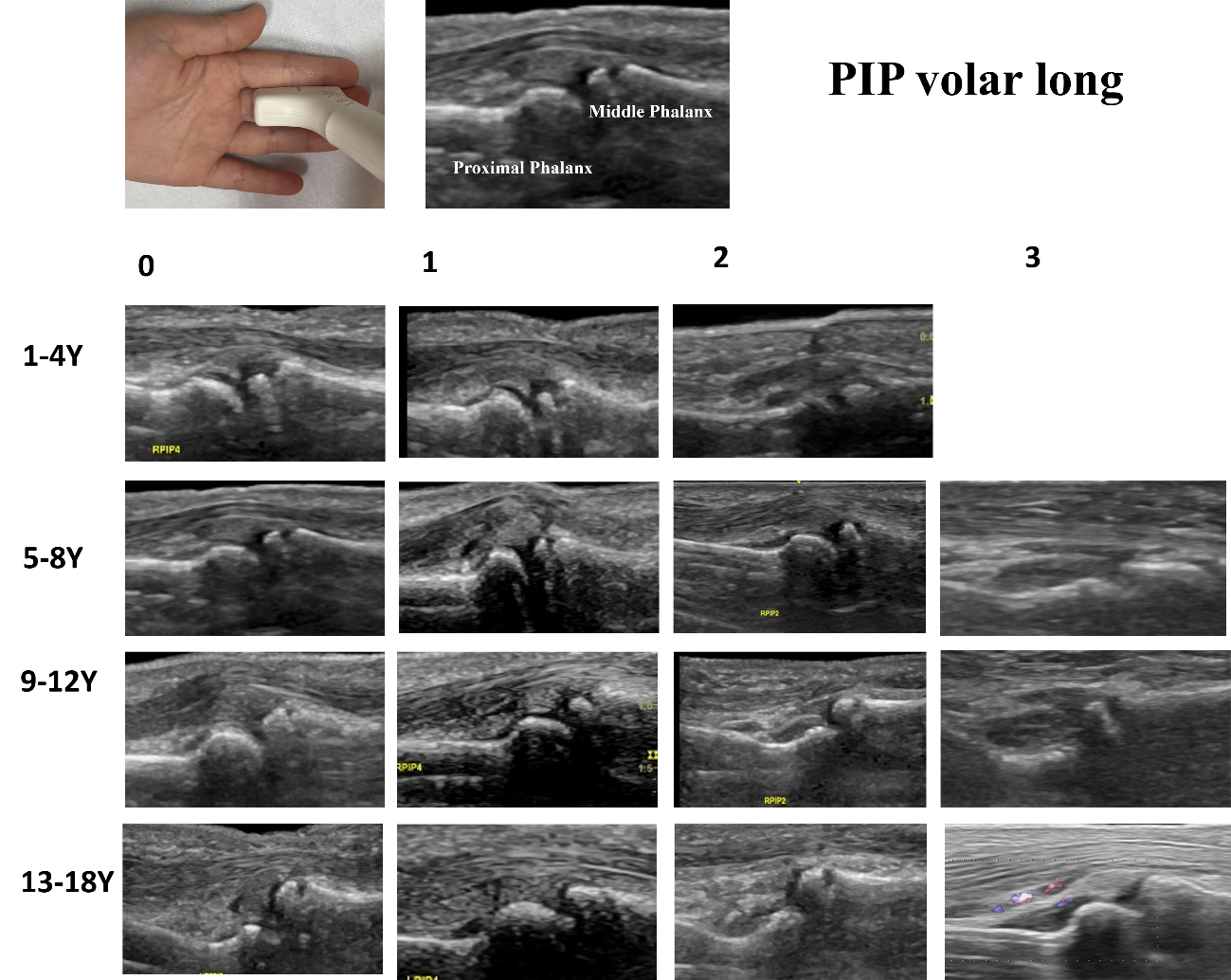 |
| Figure SB9. Ultrasound grading criteria for volar longitudinal PIP joints  Scanning method: Palm up, sagittal scanning of joint midline.  Grading criteria: Grade 0: Synovium not visible or only narrow hypoechoic band; Grade 1: Thin parallel structure; Grade 2: Proximal rounded/cystic not exceeding metacarpal head; Grade 3: Exceeding metacarpal head and elevating volar plate. |
| 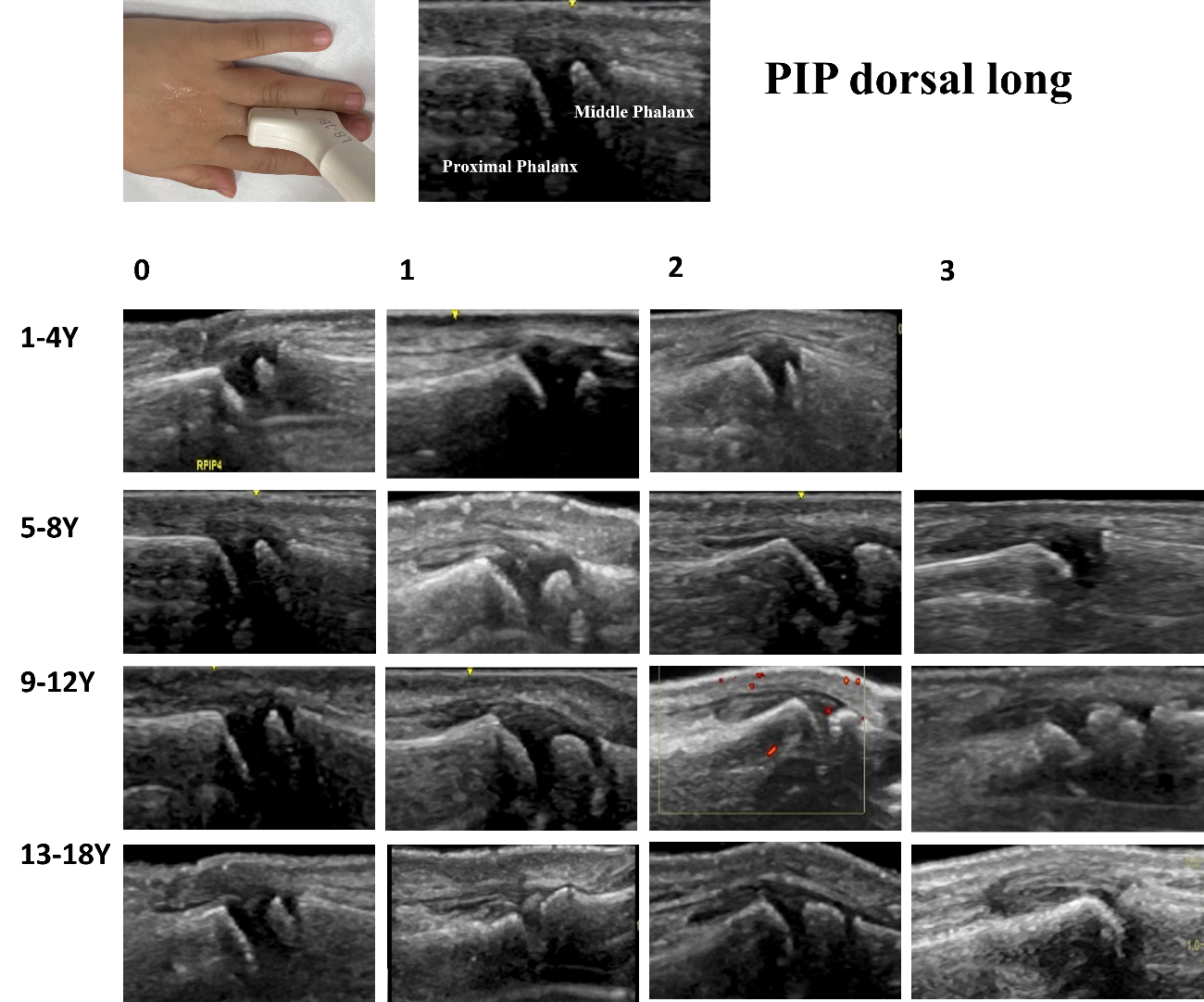 |
| Figure SB10. Ultrasound grading criteria for dorsal longitudinal PIP joints  Scanning method: Palm down, sagittal scanning of joint midline.  Grading criteria: Grade 0: Limited to lower 1/3 of fat pad; Grade 1: Not exceeding γ-line; Grade 2: Exceeding γ-line with proximal synovium parallel to metacarpal shaft, wedge-shaped; Grade 3: Exceeding γ-line and elevating tendon, rounded proximally.  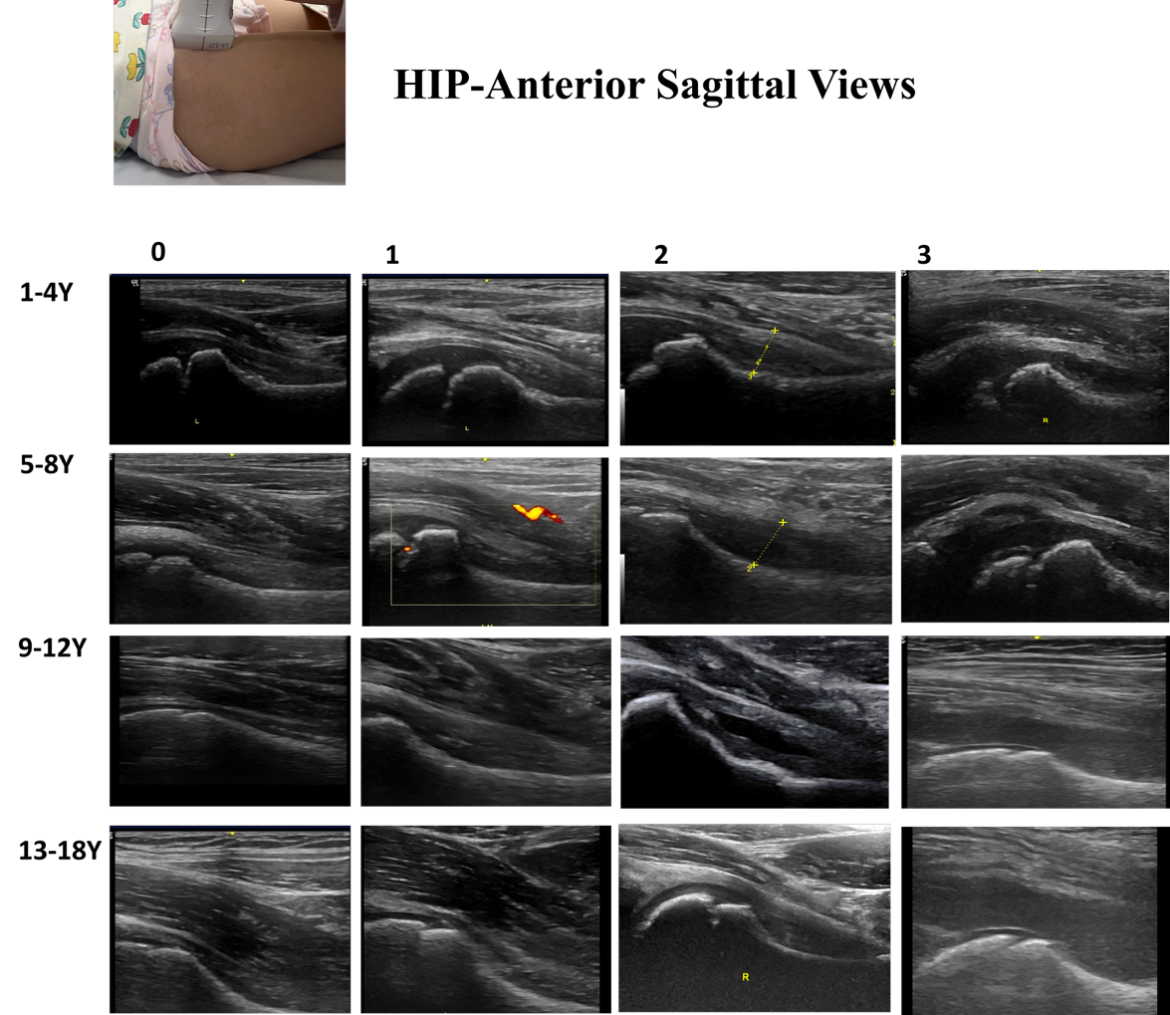  Figure SB11. Ultrasound grading criteria for hip joint  Scanning method: Supine with hip neutral and 15° external rotation, anterior sagittal scan parallel to femoral neck. Measure from femoral neck surface to outer capsular margin at maximum distension, including both anterior and posterior layers.  Grading criteria:Grade 0: Capsule parallel to femoral head/neck; bilateral difference <2.0 mm；Grade 1: Capsular distension with concave shape to femoral neck；Grade 2: Capsular distension with straight shape to femoral neck；Grade 3: Capsular distension with convex shape to femoral neck. |
| 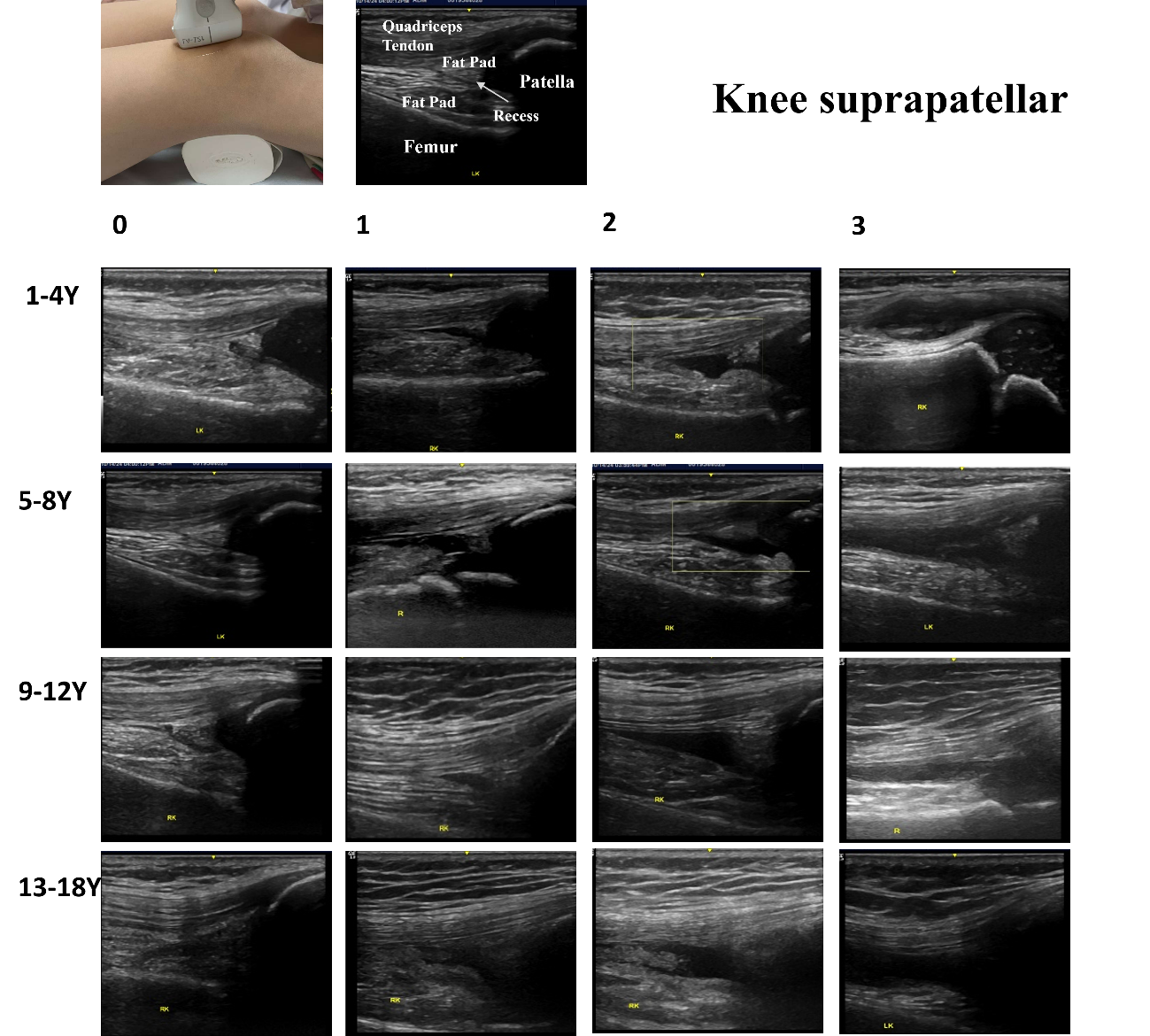 |
| Figure SB12. Ultrasound grading criteria for suprapatellar recess  Scanning method: Supine with 30° knee flexion, sagittal scanning above patella.  Grading criteria: Grade 0: No synovitis, normal or physiological slight joint space widening; Grade 1: <50% filling below quadriceps tendon; Grade 2: >50% filling below quadriceps tendon; Grade 3: Significant capsular distension. |
| 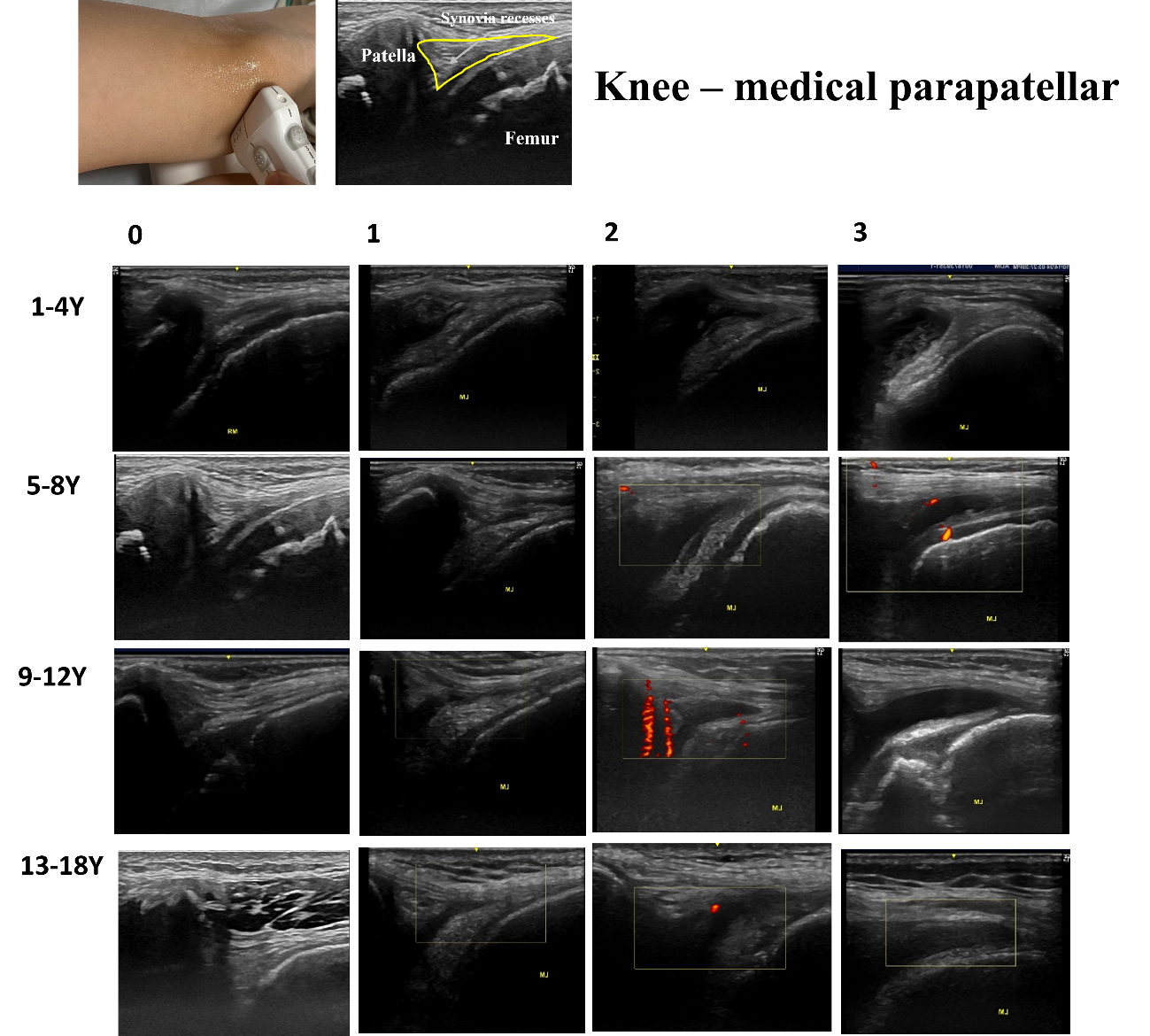 |
| Figure SB13. Ultrasound grading criteria for medial parapatellar recess  Scanning method: Transverse view, probe placed at mid-patella (or area of maximum distension), showing patella.  Grading criteria: Grade 0: No or narrow band of effusion/synovial thickening in parapatellar depression; Grade 1: Effusion/synovial thickening filling <1/3 of joint capsule; Grade 2: Filling 1/3-2/3 of joint capsule; Grade 3: Filling >2/3 of joint capsule. |
| 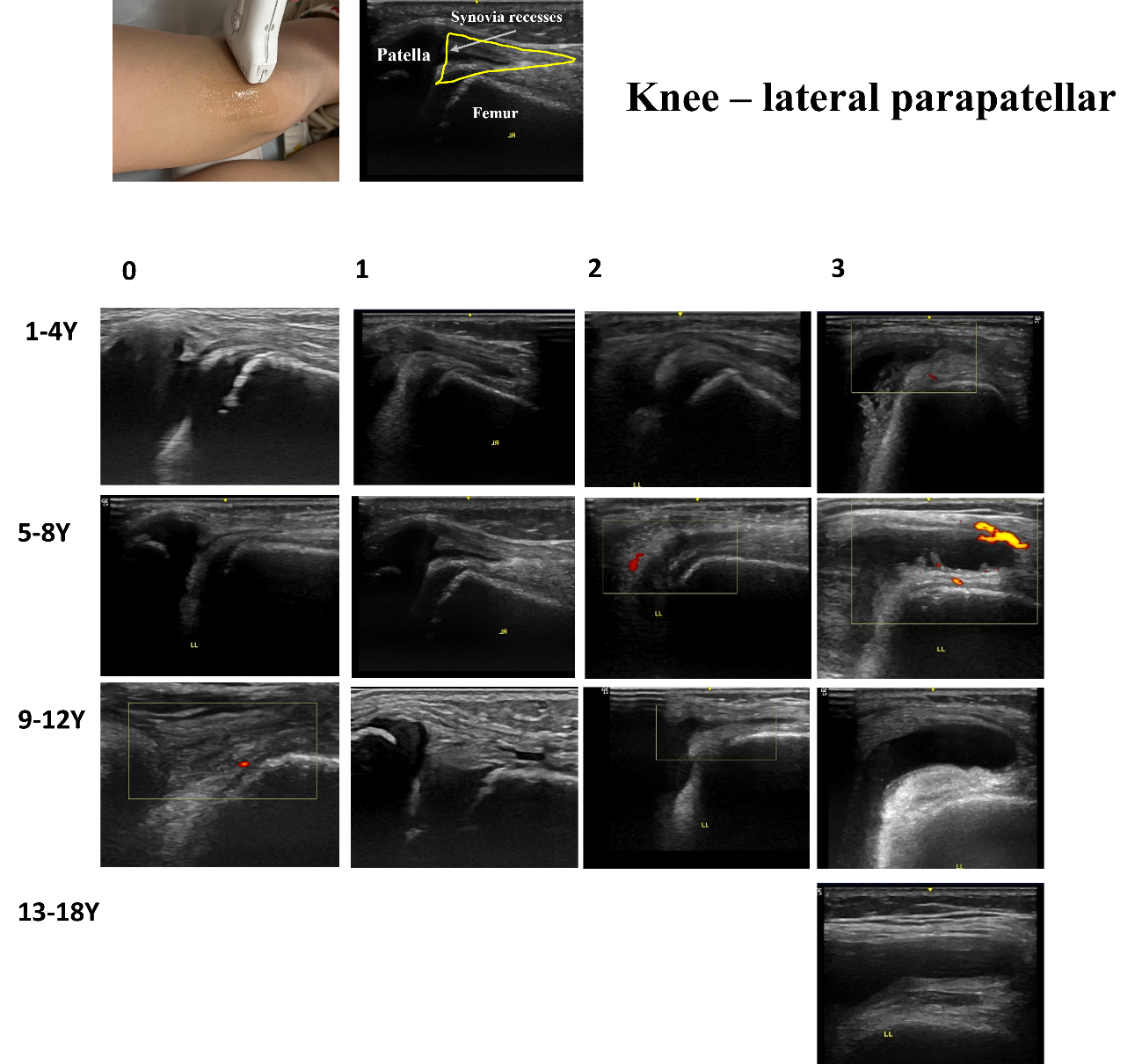 |
| Figure SB14. Ultrasound grading criteria for lateral parapatellar recess  Scanning method: Transverse view, probe placed at mid-patella (or area of maximum distension), showing patella.  Grading criteria: Grade 0: No or narrow band of effusion/synovial thickening in parapatellar depression; Grade 1: Effusion/synovial thickening filling <1/3 of joint capsule; Grade 2: Filling 1/3-2/3 of joint capsule; Grade 3: Filling >2/3 of joint capsule. |
| 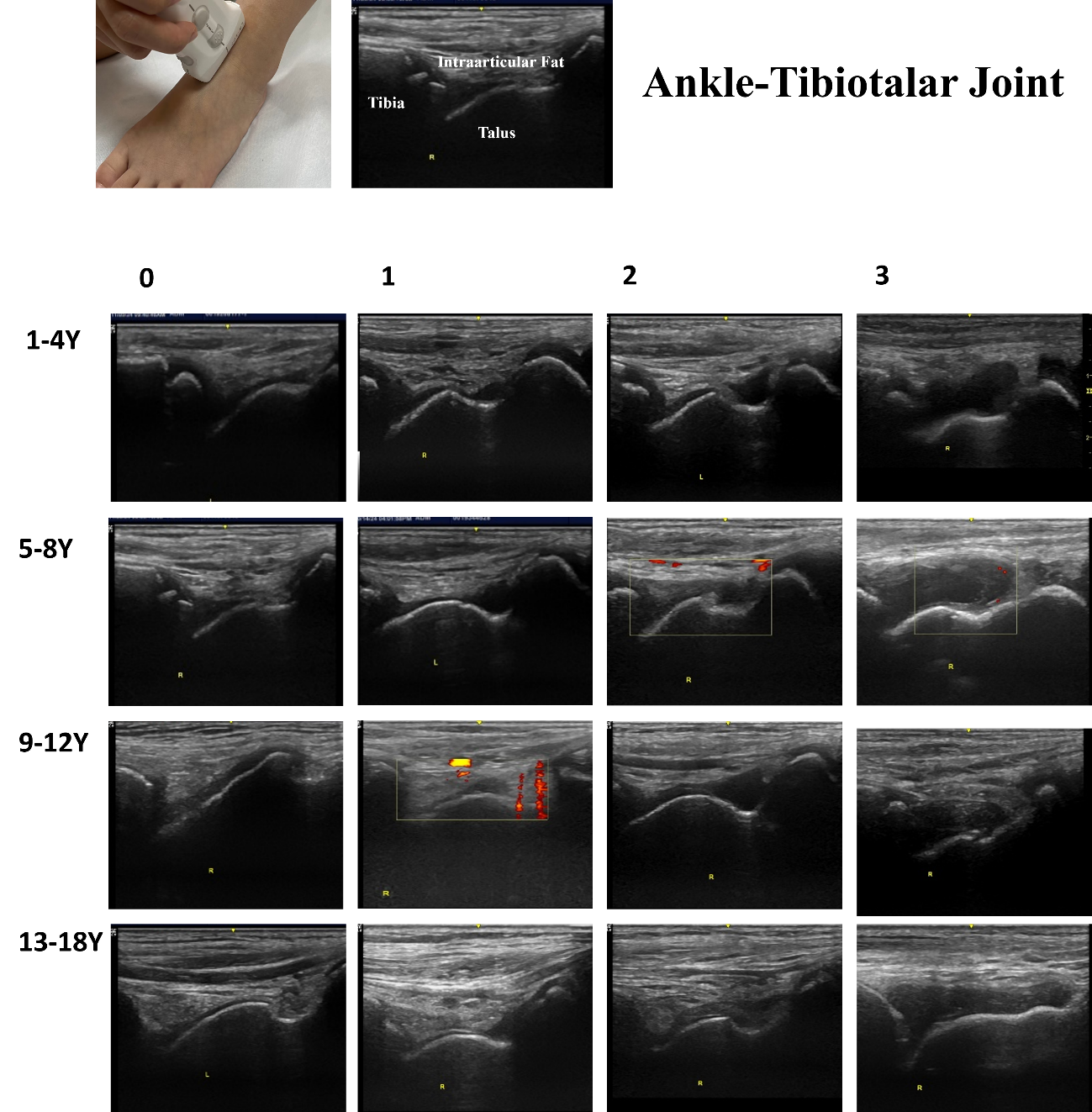 |
| Figure SB15. Ultrasound grading criteria for tibiotalar joint  Scanning method: Foot flat, sagittal scanning of anterior recess showing medial distal tibia and full length of talus.  Grading criteria: Grade 0: No effusion; Grade 1: Mild concave effusion/synovial thickening (<25% filling); Grade 2: Moderate convex effusion/synovial thickening (25%-50% filling); Grade 3: Convex filling >50%. |
| 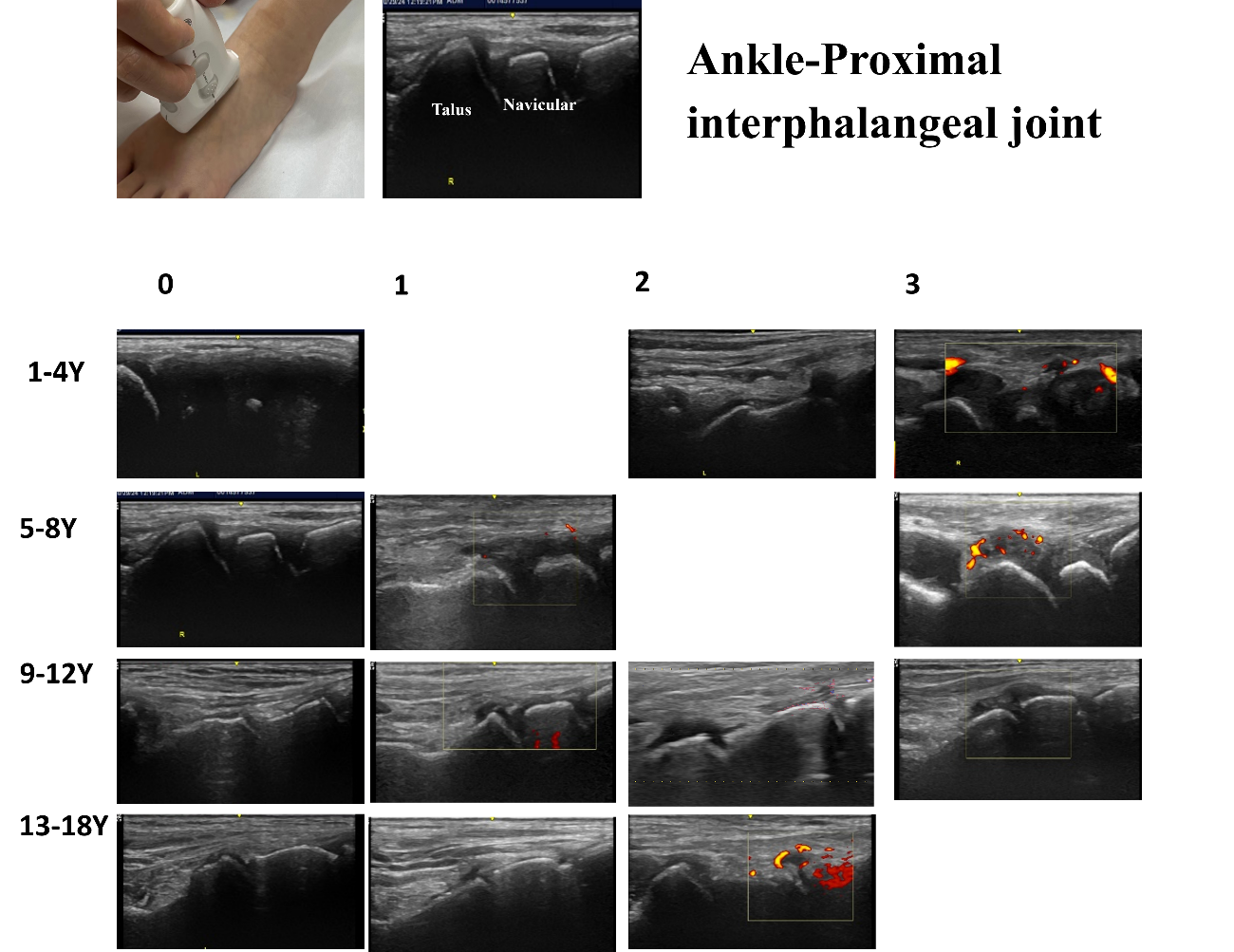 |
| Figure SB16. Ultrasound grading criteria for talonavicular joint  Scanning method: Midline longitudinal view, proximal at talus extending distally to navicular.  Grading criteria: Grade 0: Normal joint space with angular/V-shaped depression; Grade 1: Joint space widening from angular/V-shape to mild distension; Grade 2: Convex expansion to ≤50% of visible bony landmarks; Grade 3: Convex expansion to >50% of visible bony landmarks. |
| 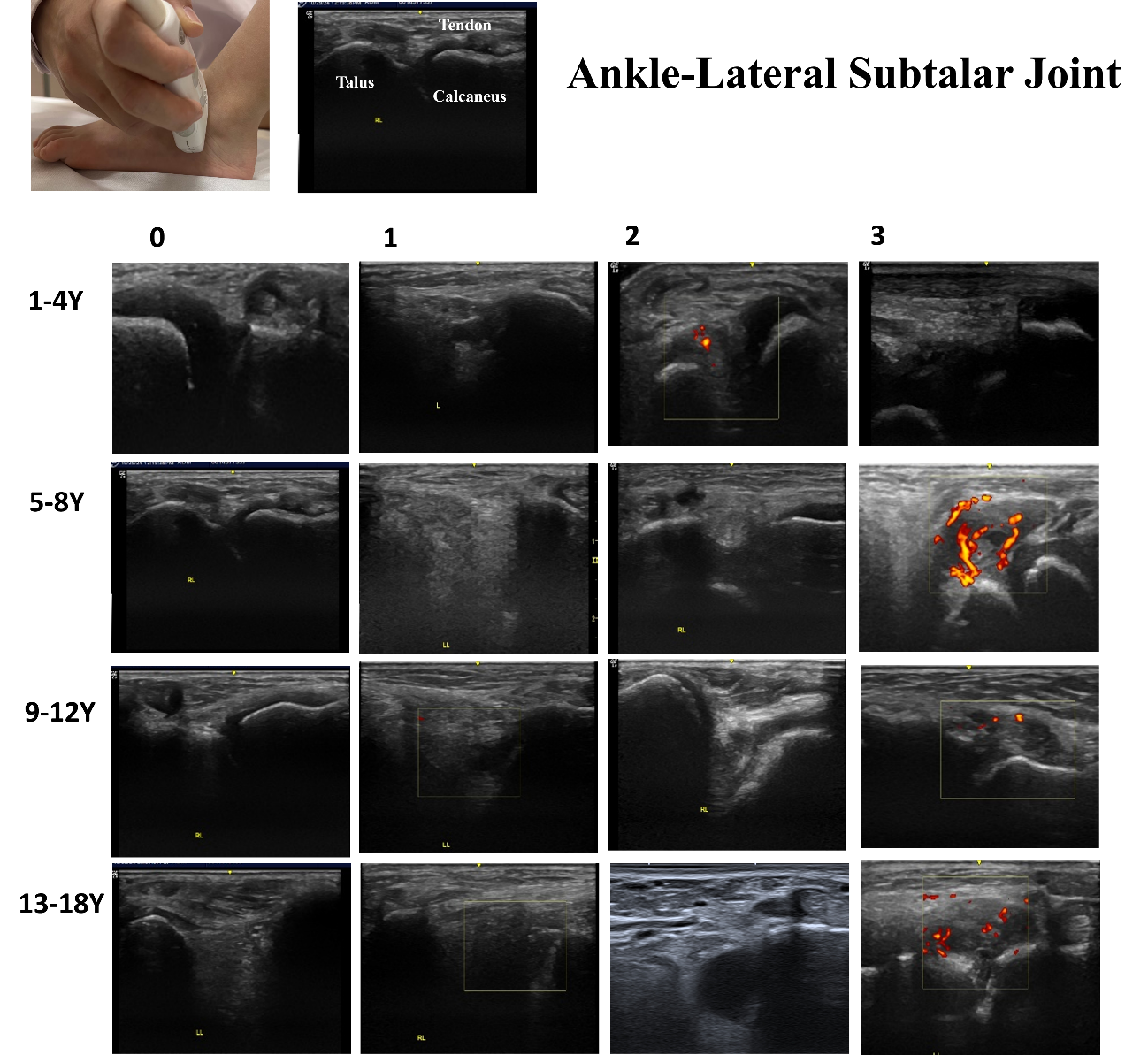 |
| Figure SB17. Ultrasound grading criteria for posterior subtalar joint  Scanning method: Lateral position, probe perpendicular to plantar surface, sliding along calcaneal sinus, showing joint space between talus and calcaneus.  Grading criteria: Grade 0: Normal joint space with angular/V-shaped depression; Grade 1: Joint space widening from angular/V-shape to mild distension; Grade 2: Convex expansion to ≤50% of visible bony landmarks; Grade 3: Convex expansion to >50% of visible bony landmarks. |
| 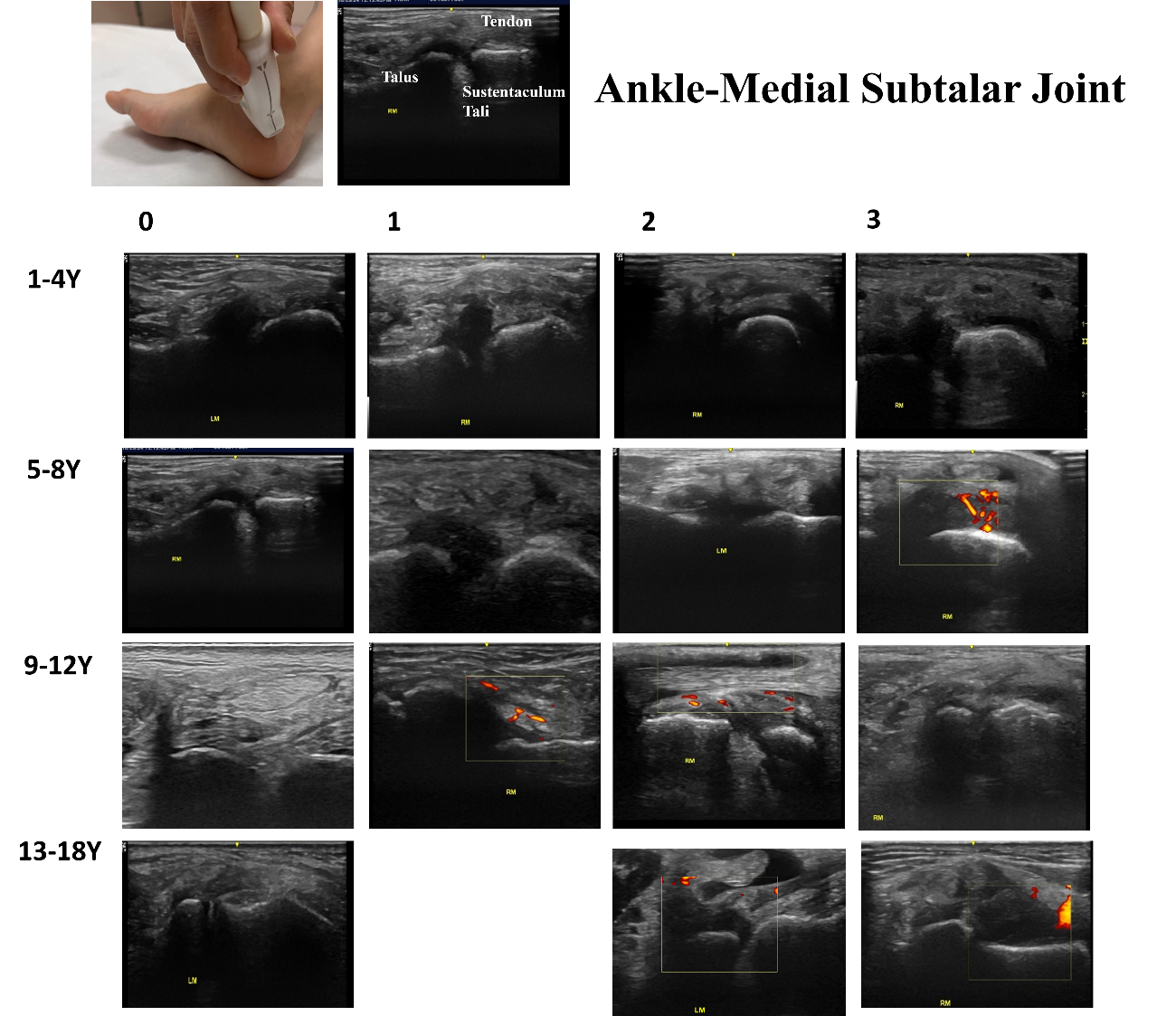 |
| Figure SB18. Ultrasound grading criteria for anterior subtalar joint  Scanning method: Medial position, probe placed anterior to medial malleolus, showing joint space between talus and subtalar support band.  Grading criteria: Grade 0: Normal joint space with angular/V-shaped depression; Grade 1: Joint space widening from angular/V-shape to mild distension; Grade 2: Convex expansion to ≤50% of visible bony landmarks; Grade 3: Convex expansion to >50% of visible bony landmarks. |
| 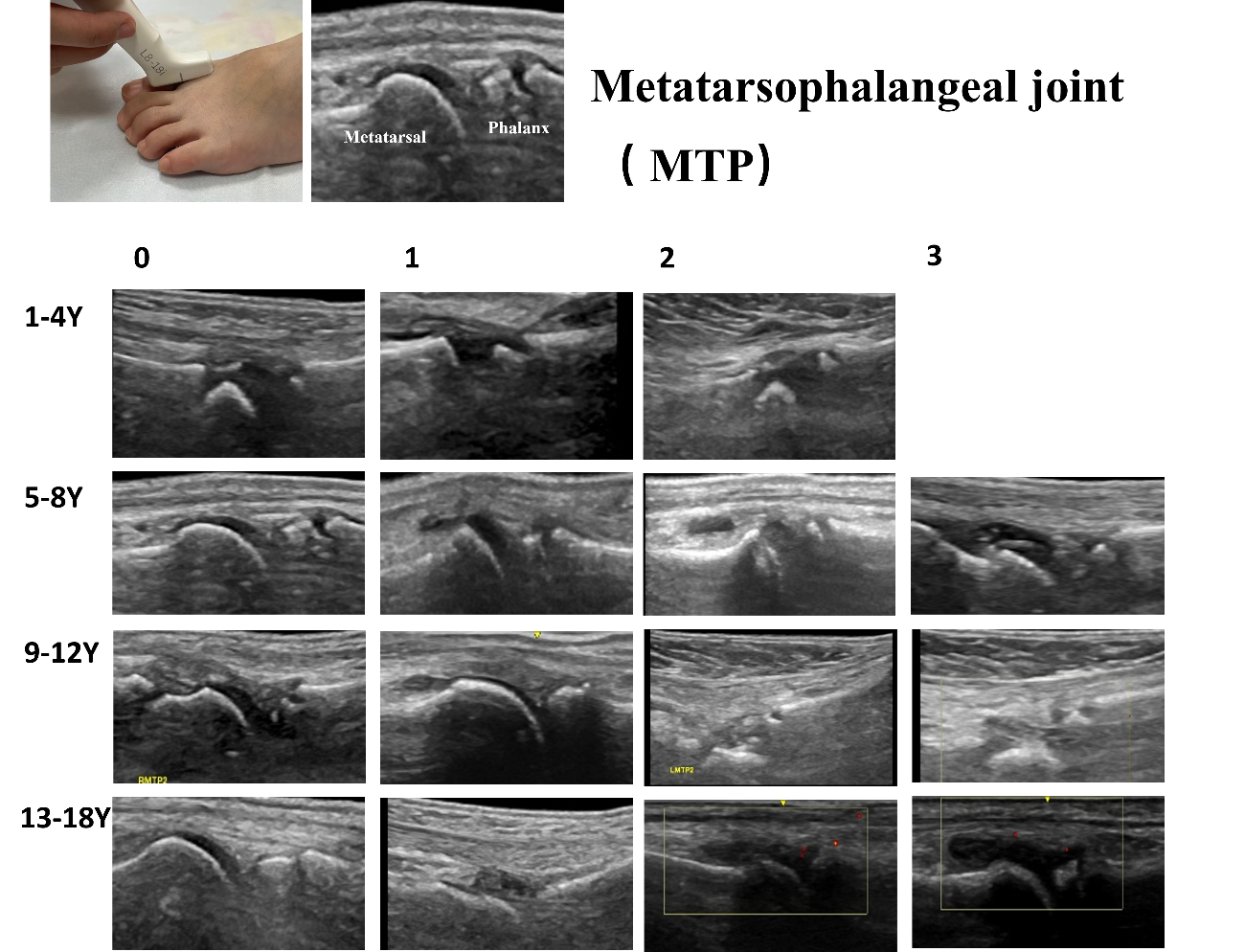 |
| Figure SB19. Ultrasound grading criteria for toe joints  Scanning method: MTP dorsal longitudinal axis, similar to finger MCP scanning method Grading criteria: Grade 0: Effusion/synovial thickening limited to lower 1/3 of joint depression; Grades 1-3 same as finger MCP grading criteria. |
| 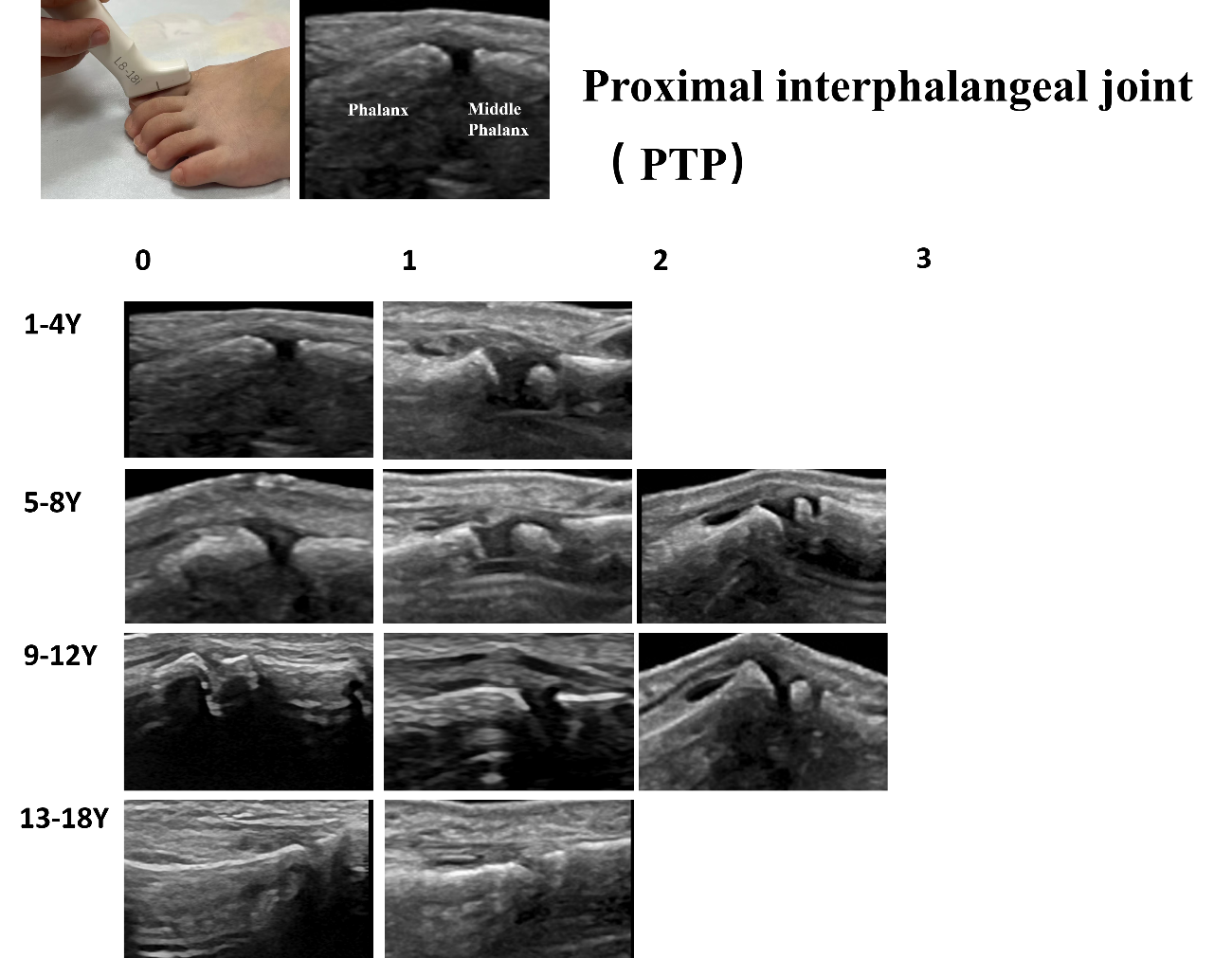 |
| Figure SB20. Ultrasound grading criteria for toe joints  Scanning method: PTPdorsal longitudinal axis, similar to finger MCP scanning method Grading criteria: Grade 0: Effusion/synovial thickening limited to lower 1/3 of joint depression; Grades 1-3 same as finger MCP grading criteria. |
| 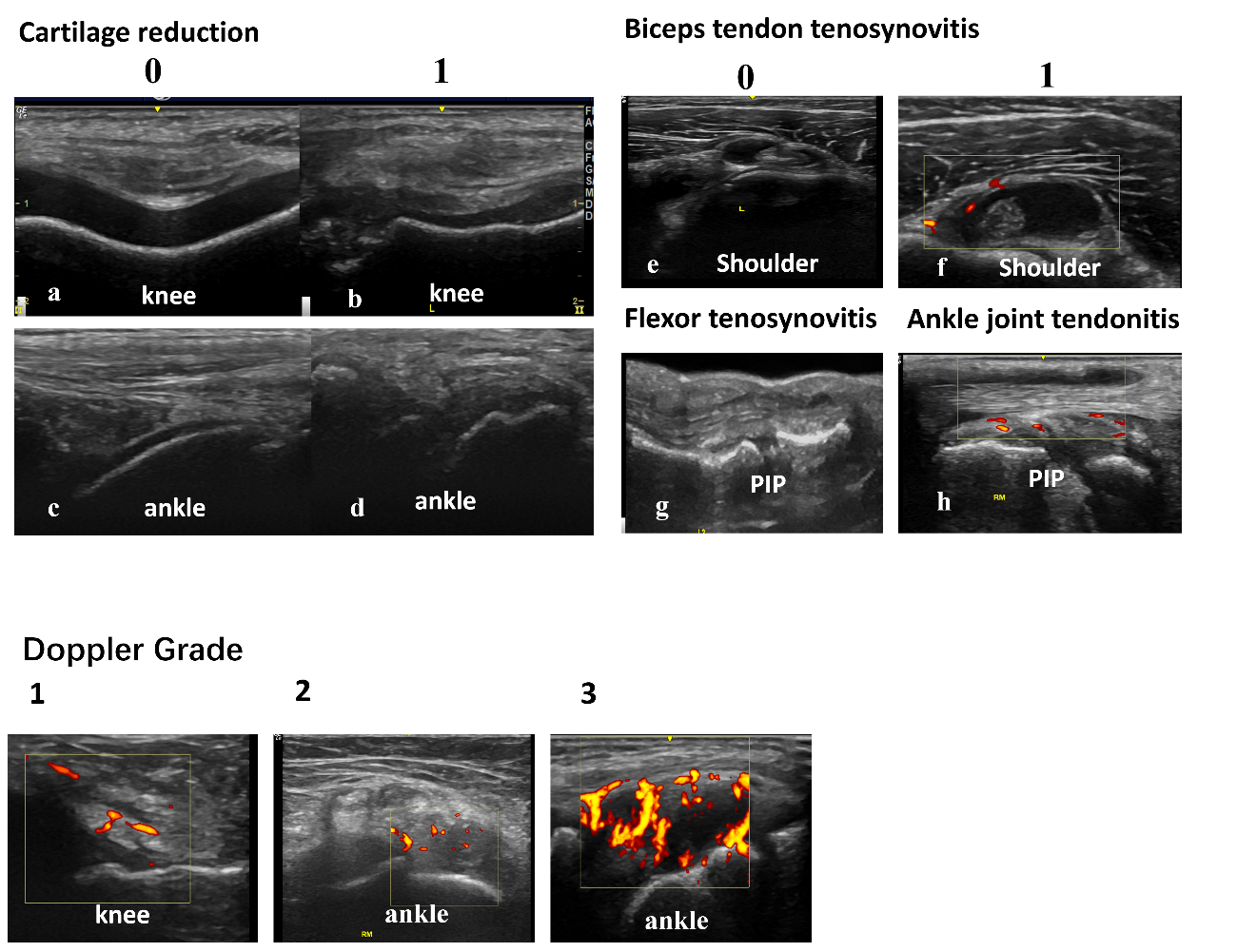 |
| Figure SB21. Ultrasound manifestations of power Doppler signals in JIA patients  Power Doppler grading (grades 1-3): Grade 1=1-3 signals, Grade 2=>3 signals or <50% confluence, Grade 3=>50% confluent signals. |
