# Supplementary material for: Development and validation of subtype-specific simplified ultrasound assessment systems for juvenile idiopathic arthritis: a prospective observational study
Source: Front Pediatr. 2026 Jul 6;14:1876983. doi: 10.3389/fped.2026.1876983 (PMC13381455; doi:10.3389/fped.2026.1876983)
Supplement: Supplementary file 3 [file Datasheet3.docx]

**Figure S1. Joint-Specific Clinical-Ultrasound Concordance by Subtype**

Heatmap visualization of clinical-ultrasound concordance across 11 joint types. Left panel: overall cohort (n=83); middle panel: oJIA subgroup (n=42); right panel: pJIA subgroup (n=41). Color scale indicates agreement level: green (moderate-substantial, Kappa>0.4), yellow (fair-slight, Kappa 0.2-0.4), orange (slight, Kappa 0.0-0.2), red (poor, Kappa<0.0). Note the marked variation across anatomical sites and between subtypes, supporting the development of subtype-specific assessment protocols.

**Figure S2. Clinical-Ultrasound Concordance Matrix by Joint Type**

Comprehensive distribution of concordant and discordant findings across all joint types. For each joint, four categories are shown: (1) US+&Clinical+ (both positive), (2) US+&Clinical- (US detects subclinical synovitis), (3) US-&Clinical+ (clinical examination more sensitive), and (4) US-&Clinical- (both negative). Left panel: overall cohort (n=83); middle panel: oJIA subgroup (n=42); right panel: pJIA subgroup (n=41). Note the predominance of US+&Clinical- findings in superficial small joints (reflecting subclinical synovitis detection) and US-&Clinical+ findings in deep joints (hip, ankle), illustrating the complementary strengths of both assessment modalities.

**Figure S3. Performance of Simplified Models at Different Disease Activity Levels**

Stacked bar charts showing the performance of simplified ultrasound assessment models at different disease activity levels. Left: Low disease activity patients; Right: High disease activity patients. From top to bottom: all patients, oligoarticular JIA, polyarticular JIA. In high disease activity states, power Doppler sensitivity significantly improved: all-patient group (78.2% to 92.6%), oligoarticular JIA group (80.8% to 93.3%), polyarticular JIA group (65.5% to 91.7%). Joint color coding as shown in legend. Sample sizes: low activity n=55, high activity n=27.

**Figure S4. Correlation Analysis of Ultrasound Parameters with Clinical Disease Activity Indicators**

Heat map showing Spearman correlation coefficients (rs) between ultrasound scoring parameters and clinical indicators in different JIA subtypes and disease activity levels. Top row: all patients of each subtype; Middle row: low disease activity patients; Bottom row: high disease activity patients. Left column: all patients (n=83); Middle column: oligoarticular JIA (n=42); Right column: polyarticular JIA (n=41). Color intensity represents correlation strength (red: positive correlation, blue: negative correlation). Clinical indicators include: JADAS27 (Juvenile Arthritis Disease Activity Score-27), CRP (C-reactive protein), ESR (erythrocyte sedimentation rate), AJC (active joint count), PhGA (physician global assessment), PGA (patient/parent global assessment). The pJIA group showed stronger correlations (with JADAS27: rs=0.68, P<0.001; with AJC: rs=0.52, P<0.01), while the oJIA group showed relatively weaker correlations. Notably, decreased correlation was observed in high disease activity pJIA patients (with JADAS27: rs=0.15-0.19, P>0.05 vs low activity: rs=0.43-0.46, P<0.01), suggesting "clinical-imaging dissociation."

**Figure S5. Decision Curve Analysis Evaluating Clinical Net Benefit**

Decision curve analysis comparing the clinical utility of different ultrasound assessment strategies at different threshold probabilities. Analysis includes: comprehensive joint GS+PD assessment (blue line), all-patient simplified model GS+PD (orange line), oligoarticular JIA-specific model GS+PD (green line), and polyarticular JIA-specific model GS+PD (red line), compared with "treat all" (dashed line) and "treat none" (dashed line) strategies. Y-axis represents net benefit, X-axis represents threshold probability. The polyarticular JIA-specific model shows positive net clinical benefit within the threshold probability range of 0.15-0.65.
