# Supplementary material for: Development and validation of subtype-specific simplified ultrasound assessment systems for juvenile idiopathic arthritis: a prospective observational study
Source: Front Pediatr. 2026 Jul 6;14:1876983. doi: 10.3389/fped.2026.1876983 (PMC13381455; doi:10.3389/fped.2026.1876983)
Supplement: Supplementary file 4 [file Datasheet4.docx]

**Table S1. Interobserver Reliability for Ultrasound Assessment: Joint-Specific Intraclass Correlation Coefficients**

| **Joint Site** | **GS ICC (95% CI)** | **PD ICC (95% CI)** |
| --- | --- | --- |
| **Overall (68 joints)** | **0.87 (0.78-0.95)** | **0.85 (0.78-0.91)** |
| Knee | 0.93 (0.89-0.95) | 0.89 (0.85-0.91) |
| Ankle | 0.90 (0.88-0.92) | 0.85 (0.79-0.90) |
| Hip | 0.89 (0.86-0.92) | 0.85 (0.80-0.88) |
| Elbow | 0.88 (0.79-0.90) | 0.87 (0.81-0.90) |
| Wrist | 0.88 (0.79-0.90) | 0.85 (0.78-0.88) |
| Shoulder | 0.87 (0.83-0.91) | 0.86 (0.81-0.89) |
| Hand/Foot small joints | 0.85 (0.78-0.88) | 0.86 (0.82-0.91) |

**Abbreviations:** GS, grayscale; PD, power Doppler; ICC, intraclass correlation coefficient; CI, confidence interval.

**Note:** Interobserver reliability was assessed by two independent evaluators with >10 years of pediatric musculoskeletal ultrasound experience. Fifty randomly selected ultrasound images from 20 patients (representing both oJIA and pJIA subtypes) were scored independently in a blinded manner. ICC values were calculated with 95% confidence intervals. ICC interpretation: 0.75-1.00 (excellent), 0.60-0.74 (good), 0.40-0.59 (fair), <0.40 (poor).

**Table S2. Patient Baseline Characteristics**

| **Characteristic** | **All Patients (n=83)** | **oJIA (n=42)** | **pJIA (n=41)** | **P value†** |
| --- | --- | --- | --- | --- |
| **Demographics** |  |  |  |  |
| Gender, male, n (%) | 32 (38.55%) | 16 (38.10%) | 16 (39.02%) | 1.000 |
| Age at enrollment (y) | 8.00 [4.50, 11.50] | 7.00 [5.00, 10.00] | 9.00 [4.00, 13.00] | 0.126 |
| Disease duration (m) | 25.00[11.00, 40.00] | 24.00[10.00, 47.00] | 26.00[12.00, 36.00] | 0.549 |
| **Disease Activity Indicators** |  |  |  |  |
| JADAS27 | 5.00 [2.50, 7.30] | 4.00 [2.00, 5.40] | 6.10 [3.00, 9.00] | 0.005 |
| AJC | 1.00 [0.00, 3.00] | 1.00 [0.00, 1.25] | 2.00 [0.75, 4.00] | 0.002 |
| ESR (mm/h) | 3.00 [2.00, 4.20] | 3.00 [2.00, 4.00] | 3.00 [1.10, 4.20] | 0.772 |
| CRP (mg/L) | 1.80 [0.90, 3.50] | 1.50 [0.85, 3.00] | 2.10 [1.00, 4.20] | 0.183 |
| **Serological Markers** |  |  |  |  |
| ANA positive, n (%) | 13 (15.66%) | 6 (14.29%) | 7 (17.07%) | 1.000 |
| RF positive, n (%) | 8 (9.64%) | 0 (0.00%) | 8 (19.51%) | 0.002 |
| **Treatment Regimens** |  |  |  |  |
| Conventional DMARDs, n (%) | 77 (92.77%) | 38 (90.48%) | 39 (95.12%) | 0.094 |
| Biologics, n (%) | 49 (59.04%) | 21 (50.00%) | 28 (68.29%) | 0.247 |
| NSAIDs, n (%) | 41 (49.40%) | 19 (45.24%) | 22 (53.66%) | 0.285 |
| **Disease Activity Stratification** |  |  |  |  |
| High activity, n (%) | 27 (32.53%) | 15 (35.71%) | 12 (29.27%) | 0.638 |
| Low activity, n (%) | 55 (66.27%) | 26 (61.90%) | 29 (70.73%) |  |
| Remission, n (%) | 1 (1.20%) | 1 (2.38%) | 0 (0.00%) |  |

†P values are for comparisons between oJIA and pJIA groups Note: JADAS27, Juvenile Arthritis Disease Activity Score-27; AJC, active joint count; ESR, erythrocyte sedimentation rate; CRP, C-reactive protein; ANA, antinuclear antibodies; RF, rheumatoid factor; DMARDs, disease-modifying antirheumatic drugs; NSAIDs, nonsteroidal anti-inflammatory drugs.

**Table S3. Distribution of Grayscale and Power Doppler Scores Across Joint Types in Juvenile Idiopathic Arthritis**

| **Cohort** | **Joint Type** | **N** | **GS Grade n (%)** | | | | **PD Grade n (%)** | | | |
| --- | --- | --- | --- | --- | --- | --- | --- | --- | --- | --- |
|  |  |  | **0** | **1** | **2** | **3** | **0** | **1** | **2** | **3** |
| Overall (n=83) | Shoulder | 166 | 163 (98.2%) | 0  (0.0%) | 1  (0.6%) | 2  (1.2%) | 164 (98.8%) | 1  (0.6%) | 1  (0.6%) | 0  (0.0%) |
|  | Elbow | 166 | 86 (51.8%) | 43 (25.9%) | 23 (13.9%) | 14 (8.4%) | 159 (95.8%) | 4  (2.4%) | 3  (1.8%) | 0  (0.0%) |
|  | Wrist | 166 | 135 (81.3%) | 14 (8.4%) | 8  (4.8%) | 9  (5.4%) | 138 (83.1%) | 15 (9.0%) | 3  (1.8%) | 10 (6.0%) |
|  | MCP | 830 | 767 (92.4%) | 13 (1.6%) | 11 (1.3%) | 39 (4.7%) | 773 (93.1%) | 10 (1.2%) | 16 (1.9%) | 31 (3.7%) |
|  | PIP | 830 | 706 (85.1%) | 59 (7.1%) | 25 (3.0%) | 40 (4.8%) | 770 (92.8%) | 12 (1.4%) | 20 (2.4%) | 28 (3.4%) |
|  | DIP | 664 | 640 (96.4%) | 8  (1.2%) | 6  (0.9%) | 10 (1.5%) | 647 (97.4%) | 6  (0.9%) | 6  (0.9%) | 5  (0.8%) |
|  | Hip | 166 | 156 (94.0%) | 3  (1.8%) | 4  (2.4%) | 3  (1.8%) | 165 (99.4%) | 1  (0.6%) | 0  (0.0%) | 0  (0.0%) |
|  | Knee | 166 | 99 (59.6%) | 37 (22.3%) | 25 (15.1%) | 5  (3.0%) | 149 (89.8%) | 13 (7.8%) | 2  (1.2%) | 2  (1.2%) |
|  | Ankle | 166 | 121 (72.9%) | 26 (15.7%) | 8  (4.8%) | 11 (6.6%) | 145 (87.3%) | 12 (7.2%) | 6  (3.6%) | 3  (1.8%) |
|  | MTP | 830 | 781 (94.1%) | 26 (3.1%) | 16 (1.9%) | 7  (0.8%) | 807 (97.2%) | 18 (2.2%) | 3  (0.4%) | 2  (0.2%) |
|  | PTP | 830 | 748 (90.1%) | 65 (7.8%) | 14 (1.7%) | 3  (0.4%) | 820 (98.8%) | 9  (1.1%) | 1  (0.1%) | 0  (0.0%) |
| oJIA (n=42) | Shoulder | 84 | 84 (100.0%) | 0  (0.0%) | 0  (0.0%) | 0  (0.0%) | 84 (100.0%) | 0  (0.0%) | 0  (0.0%) | 0  (0.0%) |
|  | Elbow | 84 | 53 (63.1%) | 19 (22.6%) | 4  (4.8%) | 8  (9.5%) | 82 (97.6%) | 2  (2.4%) | 0  (0.0%) | 0  (0.0%) |
|  | Wrist | 84 | 74 (88.1%) | 6  (7.1%) | 3  (3.6%) | 1  (1.2%) | 77 (91.7%) | 6  (7.1%) | 1  (1.2%) | 0  (0.0%) |
|  | MCP | 420 | 418 (99.5%) | 2  (0.5%) | 0  (0.0%) | 0  (0.0%) | 419 (99.8%) | 0  (0.0%) | 1  (0.2%) | 0  (0.0%) |
|  | PIP | 420 | 409 (97.4%) | 11 (2.6%) | 0  (0.0%) | 0  (0.0%) | 419 (99.8%) | 0  (0.0%) | 1  (0.2%) | 0  (0.0%) |
|  | DIP | 336 | 336 (100.0%) | 0  (0.0%) | 0  (0.0%) | 0  (0.0%) | 336 (100.0%) | 0  (0.0%) | 0  (0.0%) | 0  (0.0%) |
|  | Hip | 84 | 82 (97.6%) | 0  (0.0%) | 1  (1.2%) | 1  (1.2%) | 84 (100.0%) | 0  (0.0%) | 0  (0.0%) | 0  (0.0%) |
|  | Knee | 84 | 53 (63.1%) | 20 (23.8%) | 11 (13.1%) | 0  (0.0%) | 77 (91.7%) | 7  (8.3%) | 0  (0.0%) | 0  (0.0%) |
|  | Ankle | 84 | 63 (75.0%) | 13 (15.5%) | 3  (3.6%) | 5  (6.0%) | 76 (90.5%) | 3  (3.6%) | 3  (3.6%) | 2  (2.4%) |
|  | MTP | 420 | 412 (98.1%) | 6  (1.4%) | 2  (0.5%) | 0  (0.0%) | 419 (99.8%) | 1  (0.2%) | 0  (0.0%) | 0  (0.0%) |
|  | PTP | 420 | 392 (93.3%) | 27 (6.4%) | 1  (0.2%) | 0  (0.0%) | 419 (99.8%) | 1  (0.2%) | 0  (0.0%) | 0  (0.0%) |
| pJIA (n=41) | Shoulder | 82 | 79 (96.3%) | 0  (0.0%) | 1  (1.2%) | 2  (2.4%) | 80 (97.6%) | 1  (1.2%) | 1  (1.2%) | 0  (0.0%) |
|  | Elbow | 82 | 33 (40.2%) | 24 (29.3%) | 19 (23.2%) | 6  (7.3%) | 77 (93.9%) | 2  (2.4%) | 3  (3.7%) | 0  (0.0%) |
|  | Wrist | 82 | 61 (74.4%) | 8  (9.8%) | 5  (6.1%) | 8  (9.8%) | 61 (74.4%) | 9 (11.0%) | 2  (2.4%) | 10 (12.2%) |
|  | MCP | 410 | 349 (85.1%) | 11 (2.7%) | 11 (2.7%) | 39 (9.5%) | 354 (86.3%) | 10 (2.4%) | 15 (3.7%) | 31 (7.6%) |
|  | PIP | 410 | 297 (72.4%) | 48 (11.7%) | 25 (6.1%) | 40 (9.8%) | 351 (85.6%) | 12 (2.9%) | 19 (4.6%) | 28 (6.8%) |
|  | DIP | 328 | 304 (92.7%) | 8  (2.4%) | 6  (1.8%) | 10 (3.0%) | 311 (94.8%) | 6  (1.8%) | 6  (1.8%) | 5  (1.5%) |
|  | Hip | 82 | 74 (90.2%) | 3  (3.7%) | 3  (3.7%) | 2  (2.4%) | 81 (98.8%) | 1  (1.2%) | 0  (0.0%) | 0  (0.0%) |
|  | Knee | 82 | 46 (56.1%) | 17 (20.7%) | 14 (17.1%) | 5  (6.1%) | 72 (87.8%) | 6  (7.3%) | 2  (2.4%) | 2  (2.4%) |
|  | Ankle | 82 | 58 (70.7%) | 13 (15.9%) | 5  (6.1%) | 6  (7.3%) | 69 (84.1%) | 9 (11.0%) | 3  (3.7%) | 1  (1.2%) |
|  | MTP | 410 | 369 (90.0%) | 20 (4.9%) | 14 (3.4%) | 7  (1.7%) | 388 (94.6%) | 17 (4.1%) | 3  (0.7%) | 2  (0.5%) |
|  | PTP | 410 | 356 (86.8%) | 38 (9.3%) | 13 (3.2%) | 3  (0.7%) | 401 (97.8%) | 8  (2.0%) | 1  (0.2%) | 0  (0.0%) |

GS = Grayscale synovitis score (0-3); PD = Power Doppler signal score (0-3). N = Total number of joint examinations per joint type. Data presented as count (percentage of total examinations for that joint type). MCP = metacarpophalangeal; PIP = proximal interphalangeal; DIP = distal interphalangeal; MTP = metatarsophalangeal; PTP = proximal toe phalangeal.
