# Supplementary figures and images for: Development and validation of subtype-specific simplified ultrasound assessment systems for juvenile idiopathic arthritis: a prospective observational study
Source: Front Pediatr. 2026 Jul 6;14:1876983. doi: 10.3389/fped.2026.1876983 (PMC13381455; doi:10.3389/fped.2026.1876983)

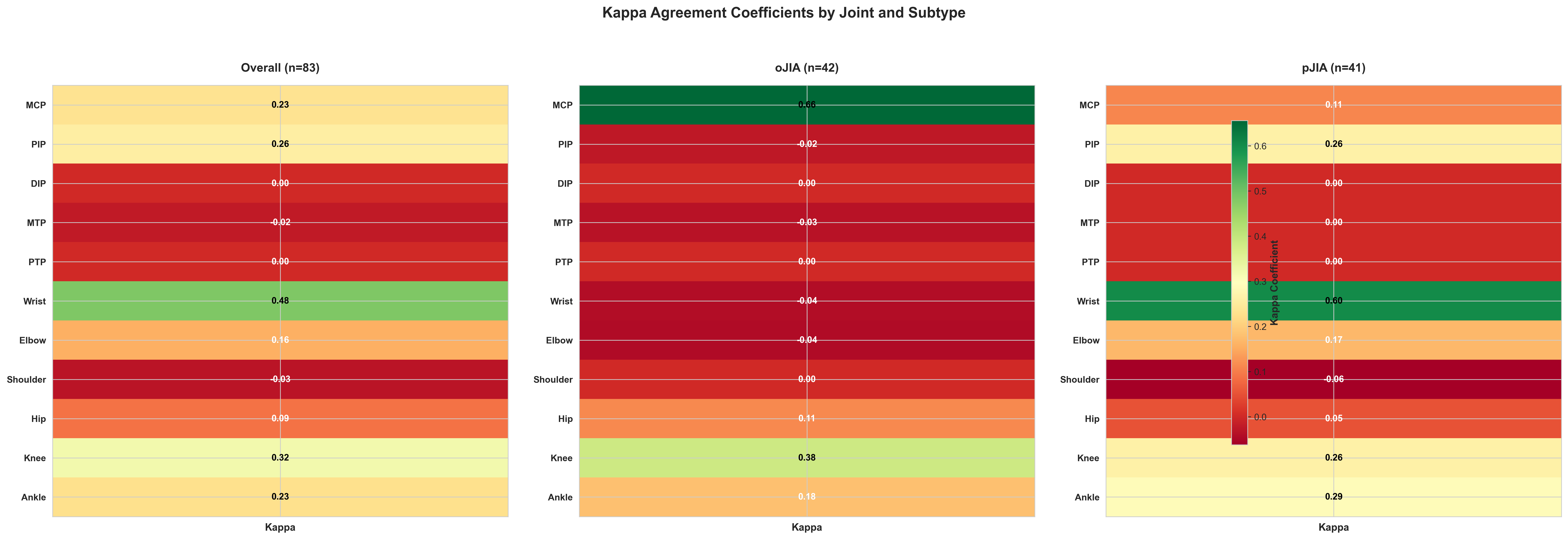

Supplement: Supplementary file 5 [file Image1.tiff]

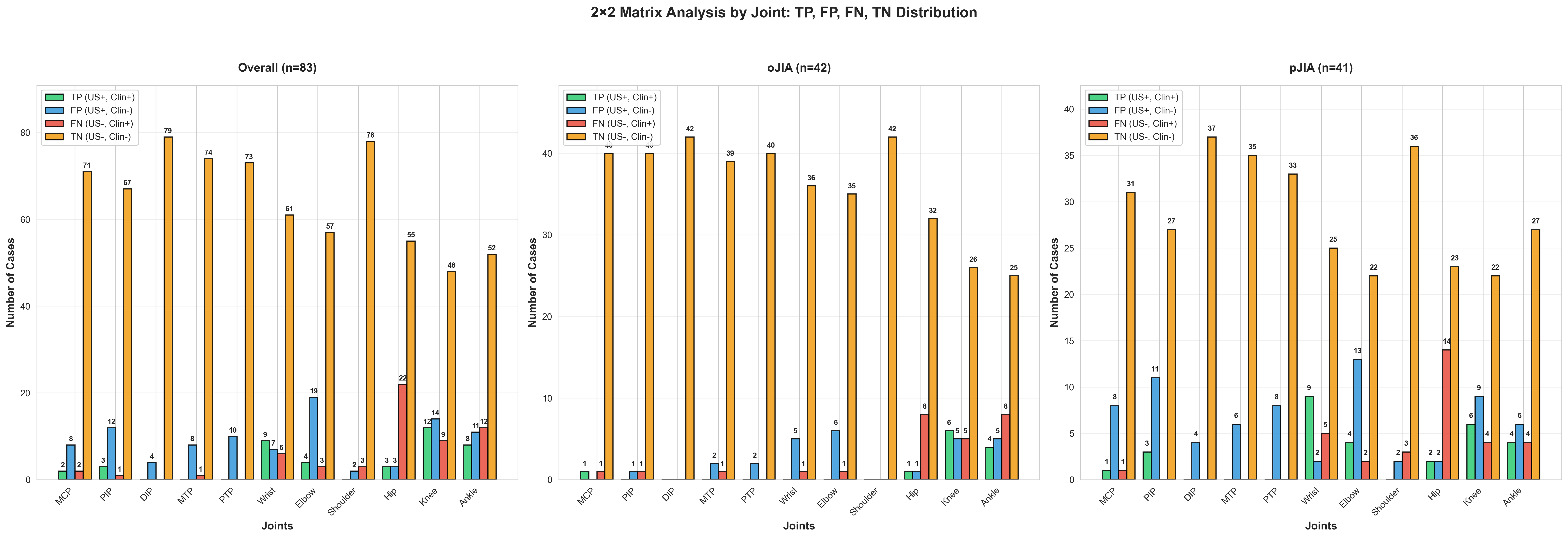

Supplement: Supplementary file 6 [file Image2.tiff]

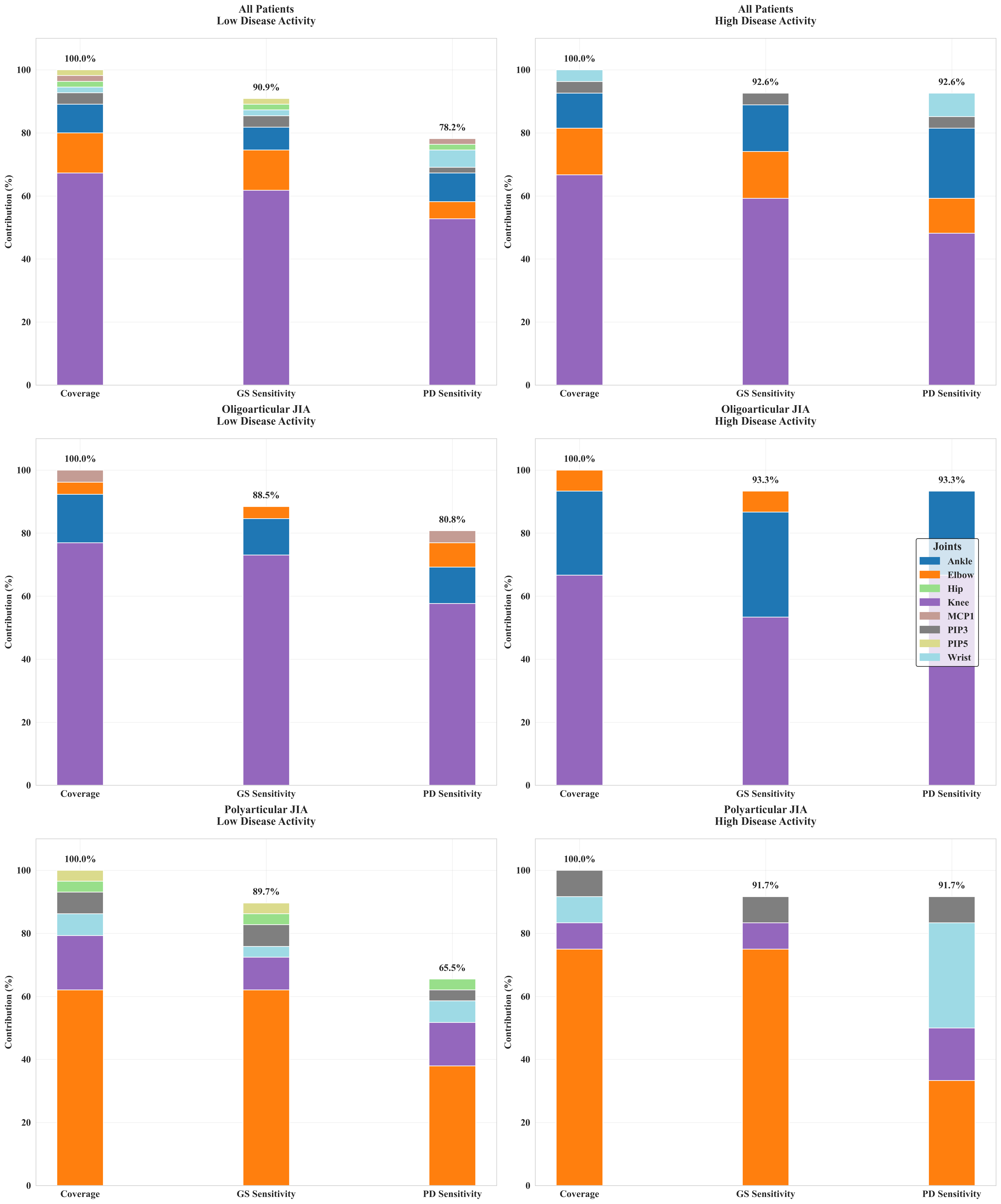

Supplement: Supplementary file 7 [file Image3.tiff]

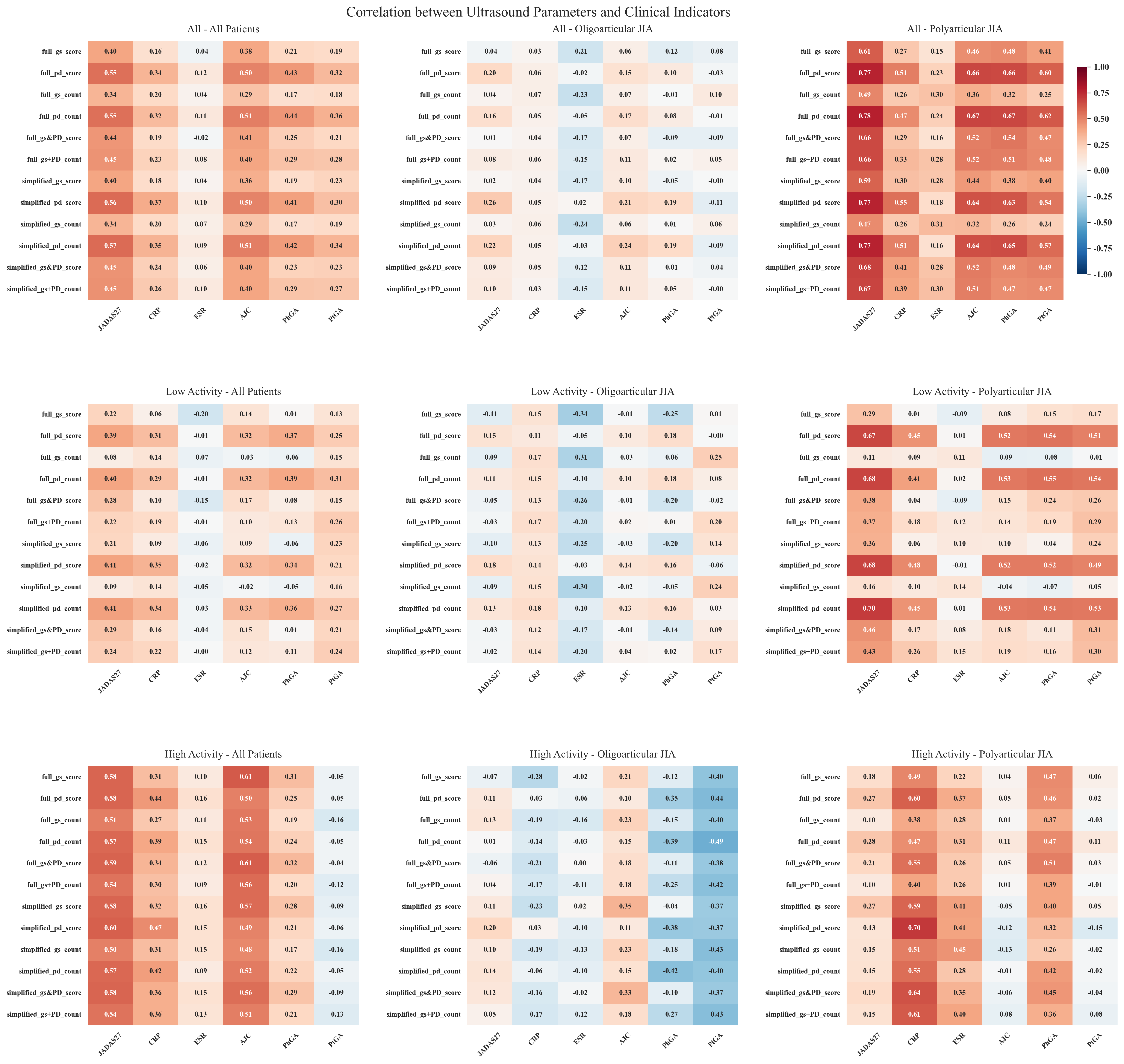

Supplement: Supplementary file 8 [file Image4.tiff]

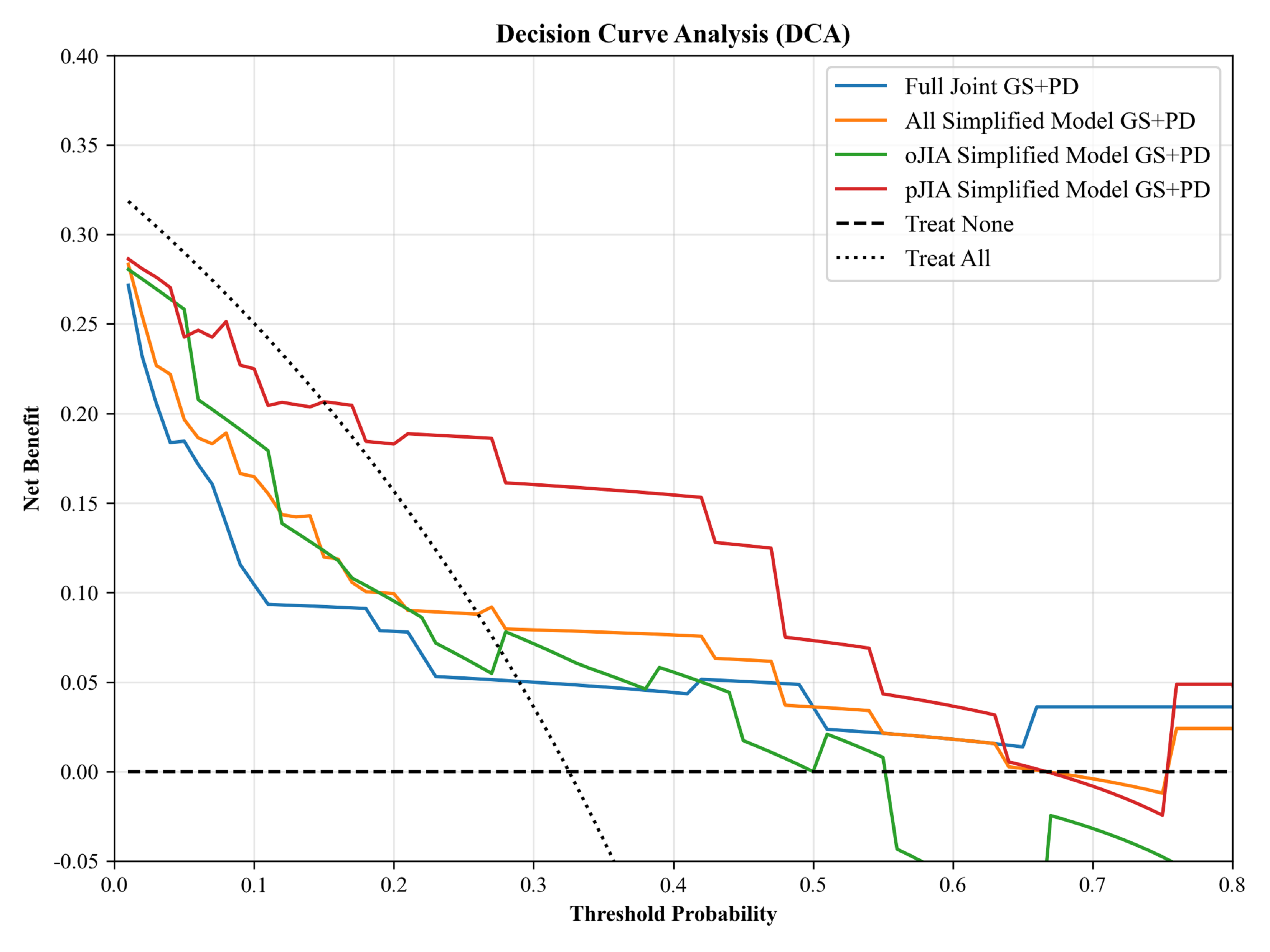

Supplement: Supplementary file 9 [file Image5.tiff]
